# Polymyxins retain *in vitro* activity and *in vivo* efficacy against “resistant” *Acinetobacter baumannii* strains when tested in physiological conditions

**DOI:** 10.1128/aac.00725-24

**Published:** 2024-09-06

**Authors:** Jennifer Rubio, Jun Yan, Sarah Miller, Jiaqi Cheng, Rachel Li, Zac Builta, Kari Aoyagi, Mark Fisher, Rosemary She, Brad Spellberg, Brian Luna

**Affiliations:** 1 Department of Molecular Microbiology and Immunology, Keck School of Medicine of USC, Los Angeles, California, USA; 2 Department of Pathology, University of Utah, Salt Lake City, Utah, USA; 3 Department of Pathology, Keck School of Medicine of USC, Los Angeles, California, USA; 4 Los Angeles General Medical Center, Los Angeles, California, USA; Bill & Melinda Gates Medical Research Institute, Cambridge, Massachusetts, USA

**Keywords:** *Acinetobacter*, polymyxins, colistin, antibiotic resistance

## Abstract

The emergence of plasmid-mediated resistance threatens the efficacy of polymyxins as the last line of defense against pan-drug-resistant infections. However, we have found that using Mueller-Hinton II (MHII), the standard minimum inhibitory concentration (MIC) medium, results in MIC data that are disconnected from *in vivo* treatment outcomes. We found that culturing putative colistin-resistant *Acinetobacter baumannii* clinical isolates, as defined by MICs of >2 mg/L in standard MHII testing conditions, in bicarbonate-containing media reduced MICs to the susceptible range by preventing colistin resistance-conferring lipopolysaccharide modifications from occurring. Furthermore, the lower MICs in bicarbonate-containing media accurately predicted *in vivo* efficacy of a human-simulated dosing strategy of colistin and polymyxin B in a lethal murine infection model for some polymyxin-resistant *A. baumannii* strains. Thus, current polymyxin susceptibility testing methods overestimate the contribution of polymyxin resistance-conferring mutations and incorrectly predict antibiotic activity *in vivo*. Polymyxins may remain a viable therapeutic option against *Acinetobacter baumannii* strains heretofore determined to be “pan-resistant.”

## INTRODUCTION

Colistin (COL) is a cationic polymyxin that is considered a drug of last resort against highly lethal, carbapenem-resistant Gram-negative bacterial pathogens, including carbapenem-resistant *Acinetobacter baumannii* (CRAB) and carbapenem-resistant *Enterobacterales* (CRE) (1). Polymyxin resistance is a serious threat as it eliminates the last-line antibiotics for the most antibiotic-resistant bacterial isolates. Newer, expensive antibiotics have decreased but not eliminated this threat in high-income countries. In lower- and lower-middle-income countries, the clinical need for colistin persists as newer and more costly antibiotics may be unavailable. Until recently, colistin resistance in *A. baumannii* had mostly been attributed to chromosomal mutations affecting colistin’s affinity to lipopolysaccharide (LPS). However, there has been significant concern that the spread of mobile colistin-resistance genes would effectively end the usefulness of polymyxins and therefore lead to widespread infections resistant to all available antibiotics [so-called “pan-drug-resistant” (PDR) infections] (2–4).

The accuracy of standard susceptibility testing for polymyxins has not been validated with respect to predicting actual efficacy in an infected host. Valid and accurate *in vitro* antimicrobial susceptibility assays are needed to identify which antibiotic(s) will be effective and may be used for the treatment of patients. To support these goals, groups such as the Clinical and Laboratory Standards Institute (CLSI) and the European Committee on Antimicrobial Susceptibility Testing (EUCAST) have developed guidelines for antimicrobial susceptibility testing to support the identification of effective antibiotics. The standard minimum inhibitory concentration (MIC) assay requires the use of the Mueller-Hinton II (MHII) medium as this medium has been shown to provide reproducible results at independent laboratories (5–7). However, relatively little attention has been devoted to determining what the optimal medium is for predicting *in vivo* outcomes (8, 9).

Our laboratory showed that *in vitro* MICs conducted in the physiologically relevant media, RPMI-1640, better predicted *in vivo* efficacy of other antibiotics than when conventional nutrient-rich MHII medium was used for susceptibility testing (10–13). In contrast to MHII, which is an undefined medium containing acid hydrolysate of casein, beef extract, starch, and water, RPMI-1640 is a defined medium containing 20 amino acids, 11 vitamins, inorganic salts, phenol red, and glucose. We also found that colistin-resistant strains, as defined by an MIC of >2 mg/L in MHII conditions, shifted to appear colistin susceptible when MIC test media were changed from MHII to RPMI-1640 (10). Other laboratories have independently shown that the bicarbonate-containing media, which is more physiologically relevant as compared to MHII alone, were also better at predicting *in vivo* outcomes for some antibiotics (9, 14–17).

Here, we show that the use of MHII media for *A. baumannii* susceptibility testing results in MIC data that are disconnected from *in vivo* treatment outcomes, such that polymyxin resistance detected in standard susceptibility testing does not result in loss of efficacy. Additionally, we show that for some strains, the substitution of MHII by RPMI-1640 culture media for polymyxin susceptibility enables enhanced accuracy for predicting *in vivo* efficacy, mediated by bicarbonate-induced changes to LPS structure in the bacteria.

## RESULTS

### Sodium bicarbonate sensitizes colistin-resistant *A. baumannii* to colistin

Having previously established that COL MICs in RPMI-1640 media were typically lower than those in MHII media for *A. baumannii* (11), we evaluated whether MHII antagonized, or rather RPMI-1640 promoted, COL activity by testing a hybrid media containing MHII and RPMI-1640 in equal parts. For these studies, we used LAC-4 ColR, a spontaneous colistin-resistant mutant of the clinical isolate LAC-4 WT (18). Whole-genome sequencing of LAC-4 ColR revealed a single point mutation in *pmrA*, a gene known to mediate colistin resistance in *A. baumannii* via LPS modifications (19). When cultured in MHII, the COL MIC of LAC-4 ColR corresponded with a colistin-resistant phenotype (MIC = >64 mg/L). However, equivalent COL MICs were observed between RPMI-1640 and hybrid media conditions for *A. baumannii* LAC-4 ColR (MIC = 2 mg/L), indicating that RPMI-1640 promoted COL activity (Table 1). In contrast, there was no difference in COL MIC for the intrinsically colistin-resistant strain *Proteus mirabilis* 10195 between MHII and RPMI-1640 culturing conditions (Table 1). There was no difference in MIC for any of the conditions tested for LAC-4 WT, the colistin-susceptible strain.

**TABLE 1:** RPMI-1640 potentiates the activity of colistin

Species	Strain	COL MIC (mg/L)			
		MHII	RPMI-1640	MHII:RPMI (50:50)	MHII + 25 mM NaHCO_3_
*A. baumannii*	LAC-4 WT	2	2	2	0.5
	LAC-4 ColR	>64	2	2	0.5
*P. mirabilis*	10195	>64	>64	>64	>64

To define which component in RPMI-1640 sensitizes the colistin-resistant *A. baumannii* strain LAC-4 ColR to colistin, we added the individual RPMI-1640 components, at the exact concentrations published by the manufacturer, into MHII and determined the COL MIC for each media formulation. Addition of 25 mM NaHCO_3_ to MHII resulted in a 256-fold decrease in the COL MIC, which corresponds to a susceptible breakpoint interpretation (MIC = 2 mg/L) (Table 1; Table S1). No other RPMI-1640 components altered MHII MICs of COL.

**Fig 1:**
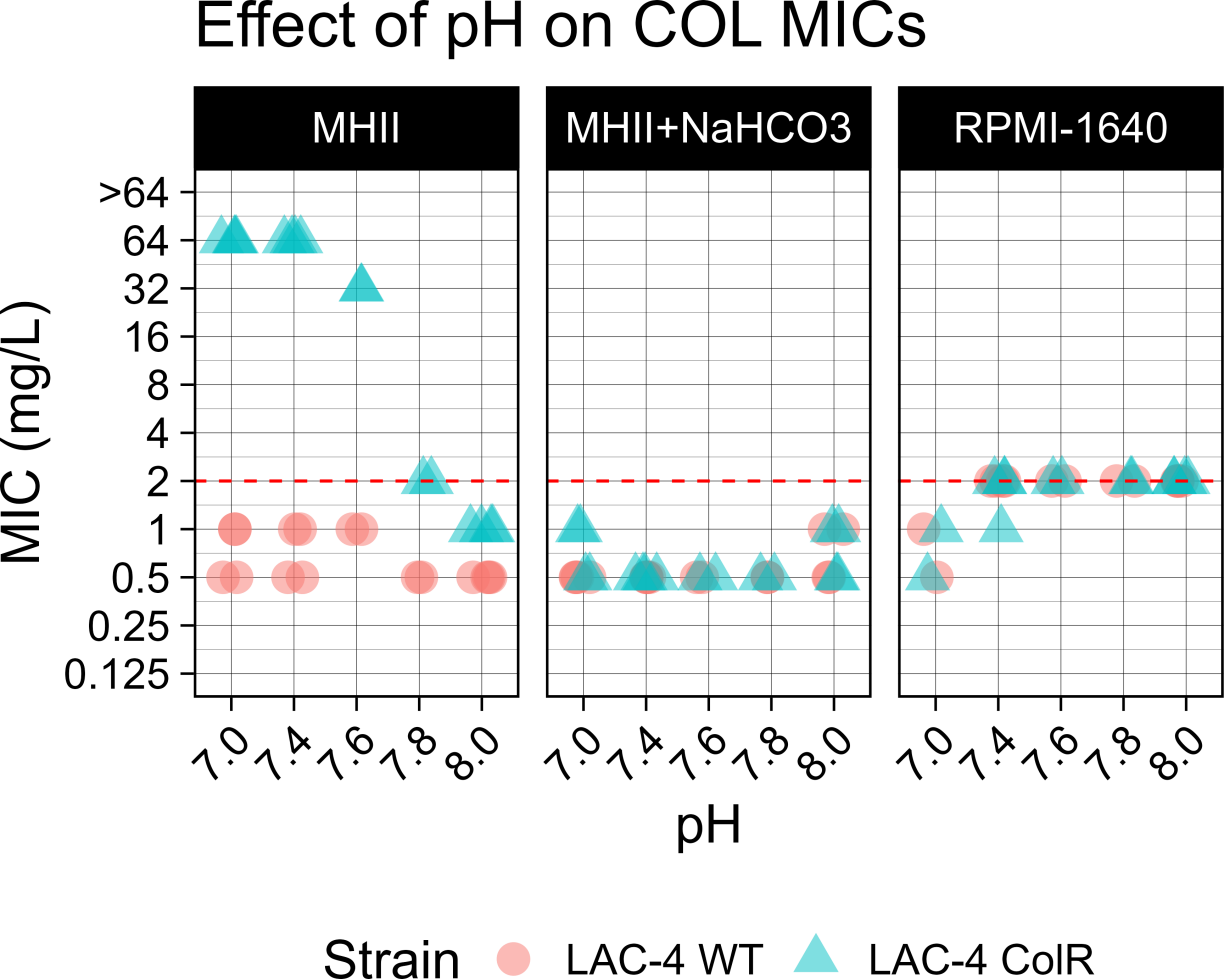
Effect of pH on colistin MICs among different media. Colistin MICs were conducted using *A. baumannii* strains LAC-4 WT (circle) and LAC-4 ColR (triangle) cultured in pH-adjusted media: MHII, RPMI-1640, and MHII + 25 mM NaHCO_3_. Media were pH adjusted using 0.1 M NaOH and HCl. Each media condition, pH, and strain were tested in duplicate. The red dashed line indicates the COL MIC breakpoint (≤2 mg/L).

To determine that the COL MIC shift in susceptibility was due to the presence of bicarbonate and not a consequence of pH change, a feature that has been previously reported to affect polymyxin activity (15), we determined COL MICs using pH-adjusted RPMI-1640, MHII, and MHII + NaHCO_3_. For the LAC-4 WT strain, no change in colistin susceptibility was observed with changes in pH (Fig. 1). Similarly, for the LAC-4 ColR strain, there was no change in COL MICs in either RPMI-1640 or MHII + NaHCO_3_ at any of the pH values tested. However, the COL MIC of LAC-4 ColR shifted to a colistin-susceptible phenotype (MIC = 2 mg/L) in MHII at the supraphysiological pH of ≥7.8 (Fig. 1).

### Bicarbonate effect on polymyxin-resistance mechanisms

**Fig 2:**
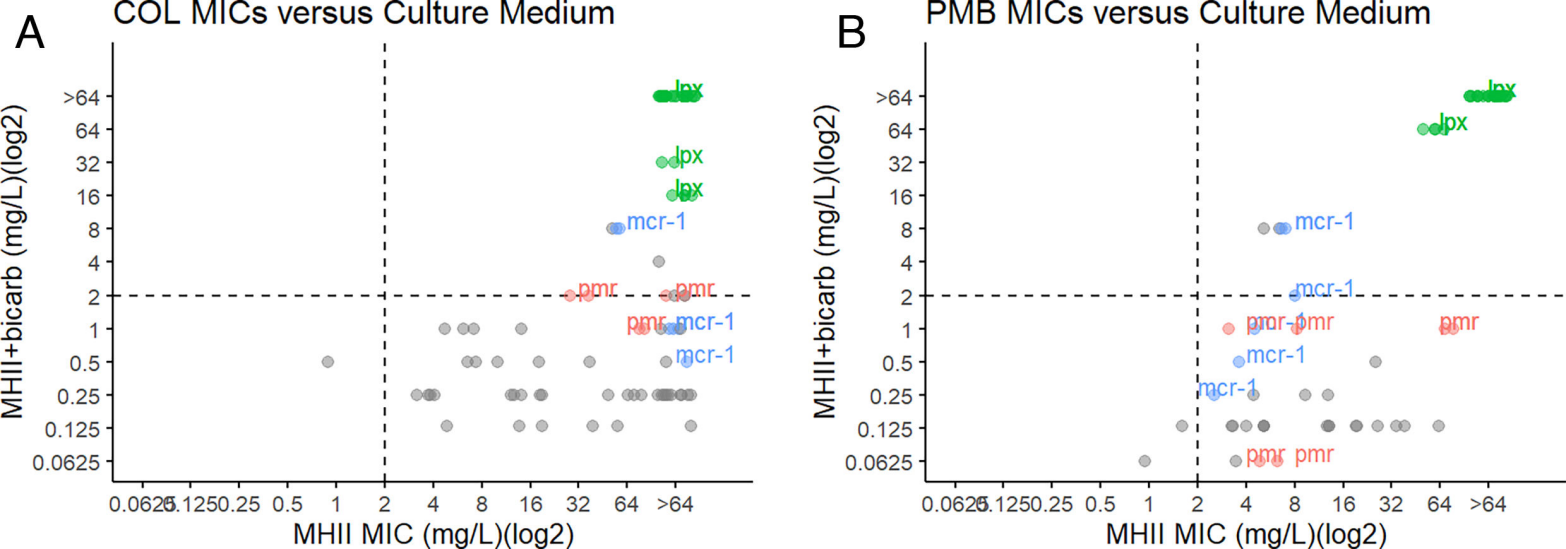
Polymyxin MICs of ColR isolates in MHII or MHII supplemented with sodium bicarbonate. (**A**) Colistin resistance is defined as MIC >2 mg/L in MHII conditions. COL MICs were conducted in MHII or MHII + NaHCO_3_ for *A. baumannii* (*n* = 47) colistin-resistant isolates with both known (*mcr-1*+ in red and *pmr* in blue) and unknown (gray) colistin resistance-conferring mutations. (**B**) Polymyxin B (PMB) resistance is defined as MIC >2 mg/L in MHII conditions. PMB MICs for colistin-resistant strains of *A. baumannii* cultured in MHII were plotted against PMB MICs conducted in MHII + 25 mM NaHCO_3_. PMB-resistant strains with known mutations included mutations in the genes *lpxA*, *lpxC*, and *lpxD* (*n* = 13) and mutations in the genes *pmrA*, *pmrB*, and *pmrC* (*n* = 3) or were positive for the *mcr-1* gene (*n* = 3). PMB resistance-conferring mutations were unknown for the remaining *A. baumannii* isolates (*n* = 16). Dashed lines indicate susceptibility breakpoint (>2 mg/L). MICs in the top right quadrant indicate values that would be considered resistant in both media conditions. MICs in the bottom right quadrant indicate values that would be considered resistant in normal MHII but susceptible in bicarbonate-containing media.

We tested a panel of clinical isolates with known and unknown colistin resistance-conferring mutations. Three isolates contained mutations in *pmrA*, *pmrB*, or *pmrC*; 3 isolates contained the mobile colistin-resistance gene (*mcr-1*); and 13 contained mutations in the genes *lpxA*, *lpxC*, and *lpxD*. With the exception of ATCC 17978 (*mcr-1*), which maintained a colistin-resistant phenotype, all other colistin-resistant *A. baumannii* strains positive for *mcr-1* or with mutations in *pmrA*, *pmrB*, or *pmrC* regained susceptibility to colistin when cultured in MHII containing bicarbonate (MIC ≤2 mg/L) (Fig. 2; Table S2). However, LPS-deficient *A. baumannii* isolates with mutations in *lpxA*, *lpxC*, *or lpxD* maintained a colistin-resistant phenotype in both MHII and MHII + NaHCO_3_ culturing conditions (Fig. 2). Interestingly, the LPS-deficient mutant strains could not be cultured in RPMI-1640. Thirty-nine of 42 colistin-resistant *A. baumannii* isolates, all of which had unknown colistin resistance-conferring mechanisms, regained susceptibility when cultured in bicarbonate-containing culturing conditions.

Next, we tested the effect of bicarbonate culturing conditions on polymyxin B (PMB) MICs of *A. baumannii* isolates with both known and unknown polymyxin resistance-conferring mutations. Similar to the COL MIC data, with the exception of ATCC 17978 (mcr-1), isolates containing mutations in *pmrA*, *pmrB*, or *pmrC* or *mcr-1*+ regained susceptibility to PMB when cultured in MHII + NaHCO_3_ (Fig. 2). Thirteen isolates containing mutations in *lpxA*, *lpxC*, or *lpxD* maintained resistance in bicarbonate conditions. Of the 17 isolates containing unknown PMB resistance-conferring mutations, 16 regained PMB susceptibility when MHII + NaHCO_3_ was used as the MIC culturing media.

### Colistin efficacy in human blood

**Fig 3:**
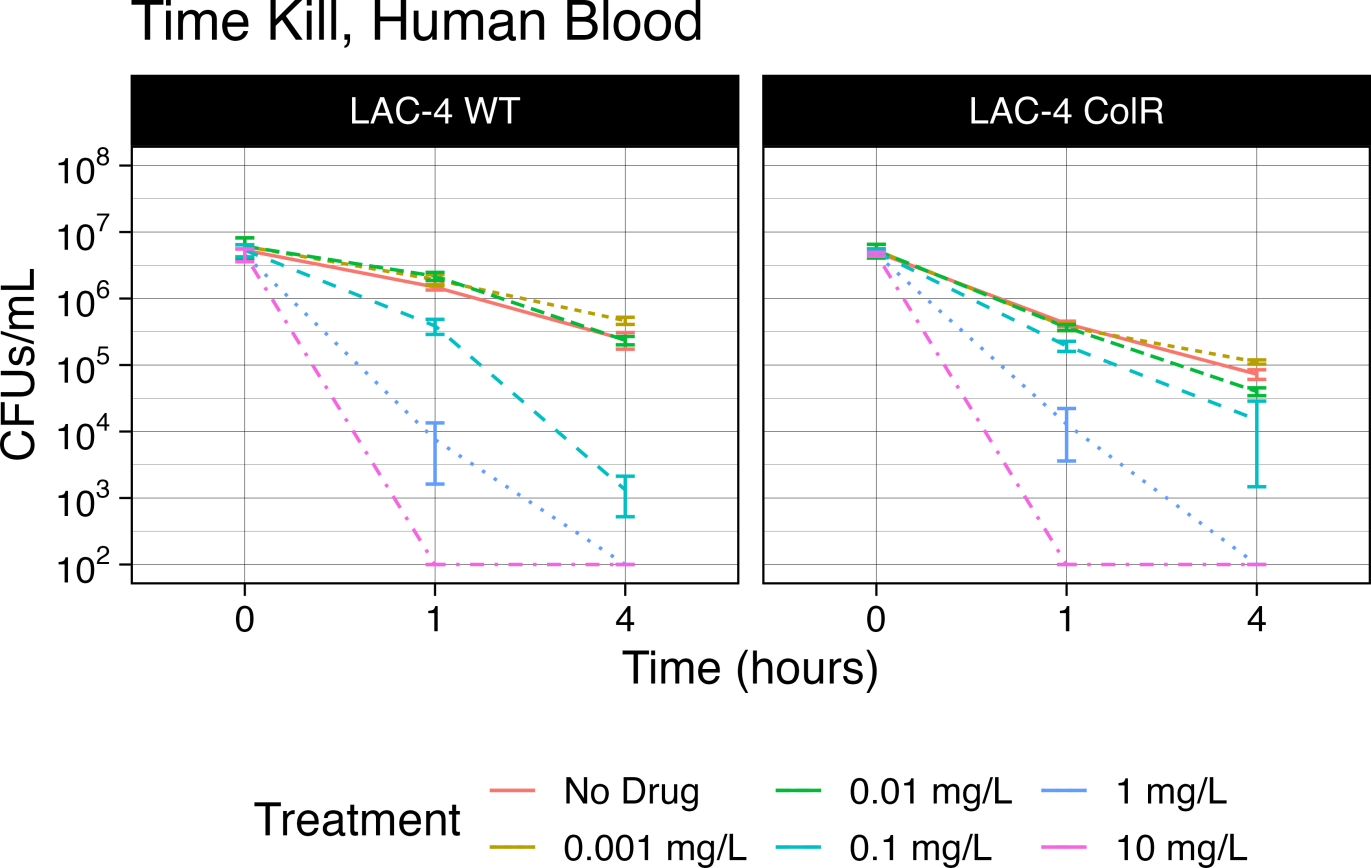
Colistin efficacy against LAC-4 in human blood culturing conditions. LAC-4 WT (WT) and LAC-4 ColR (ColR) were cultured in human blood containing the anticoagulant K2 EDTA and treated with colistin at 0.001, 0.01, 0.1, 1.0, and 10.0 mg/L. Colistin efficacy was measured via CFU per milliliter at 0, 1, and 4 h post incubation with *A. baumanni*i LAC-4 WT and ColR strains. A no-treatment control was included at each time point. Samples were serially diluted using the drop plate method, and CFUs were counted following incubation at 37°C.

The concentration of bicarbonate in RPMI-1640 is physiologically similar to human blood. Therefore, we performed time kill assays to determine if the increased activity of COL in bicarbonate-containing media would translate to effective killing in human whole blood. For the LAC-4 WT strain, the addition of 0.1-mg/L colistin to LAC-4 WT cultured in blood resulted in a significant decrease of CFUs at both 1 and 4 h post incubation compared to the no-drug control group (Mann-Whitney; *P* = 0.0020 and *P* = 0.0020, respectively) (Fig. 3). These time kill results would be predicted by the COL MICs of ≤2 mg/L in MHII and MHII + NaHCO_3_ media. When LAC-4 ColR was cultured in blood with 0.1-mg/L colistin, there was a significant decrease in CFUs at both 1 and 4 h post incubation compared to the no-drug control (Mann-Whitney; *P* = 0.0002 and *P* = 0.0079, respectively). However, these time kill results are consistent only with the MICs determined in MHII + NaHCO_3_ and are inconsistent with MICs determined using standard MHII conditions.

### Bicarbonate affects LPS structure

**Fig 4:**
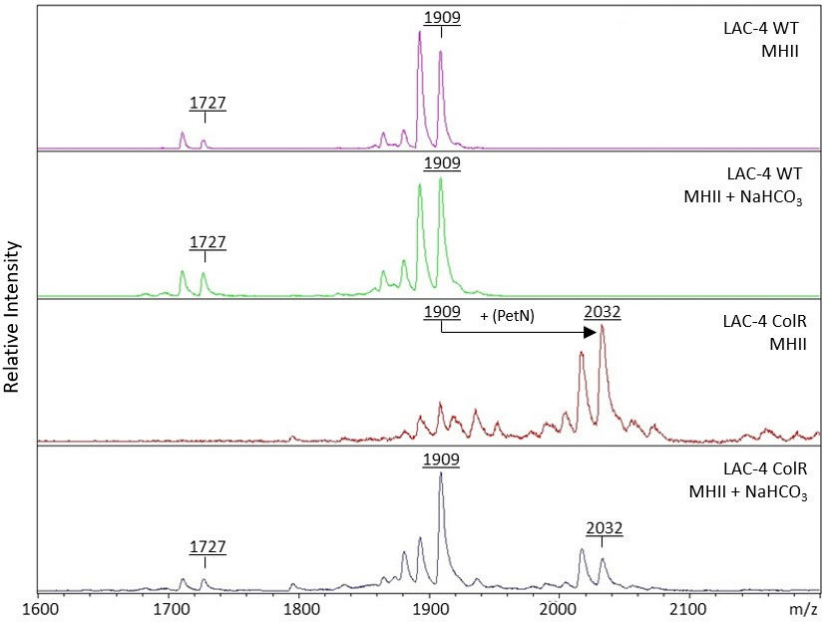
Mass spectrometry of lipid A isolated from *A. baumannii* strains LAC-4 WT and LAC-4 ColR cultured in MHII or MHII + NaHCO_3_. *A. baumannii* strains LAC-4 WT and LAC-4 ColR were cultured to mid-log phase in MHII or MHII + 25 mM NaHCO_3_. Lipid A was isolated from LAC-4 WT and LAC-4 ColR, followed by MALDI-TOF mass spectrometry in the negative-ion mode.

Using matrix-assisted laser desorption/ionization time-of-flight (MALDI-TOF) mass spectrometry, we characterized changes in LPS structure when clinical strains of *A. baumannii* were cultured in MHII or MHII + NaHCO_3_ (Fig. 4). For LAC-4 WT, the colistin-susceptible *A. baumannii* isolate, peaks at *m*/*z* values of 1,727 and 1,909, corresponding to *bis*-phosphorylated hepta- and hexa-acylated lipid A species, respectively, were similarly abundant in both MHII and MHII + NaHCO_3_ conditions. Next, we looked at the changes in LPS structure of LAC-4 ColR, a colistin-resistant *A. baumannii* isolate, when cultured in MHII or MHII + NaHCO_3_. The LAC-4 ColR strain had an additional peak at an *m*/*z* of 2,032, which is consistent with the expected spectra of LPS modified with a phosphoethanolamine (PetN, +123 *m*/*z*) group. The presence of a PetN group when LAC-4 ColR was cultured in MHII is consistent with our whole-genome sequencing, which revealed a single-point mutation in *pmrA*, a gene shown to mediate colistin resistance through addition of PetN groups to the LPS (19). The relative abundance of this peak was greatly reduced in MHII + NaHCO_3_ as compared to MHII, a pattern that is consistent with the increased susceptibility observed in this same medium.

**Fig 5:**
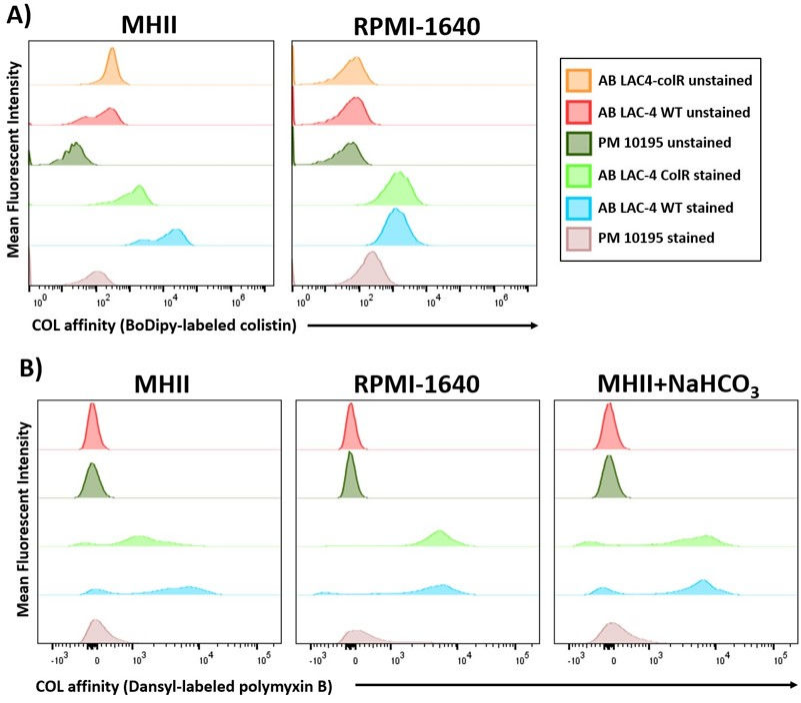
Affinity of fluorescently labeled colistin with bacteria. (**A**) *A. baumannii* LAC-4 WT or LAC-4 ColR was cultured to mid-log phase in MHII or RPMI-1640. Following incubation with BoDipy-labeled colistin (0.1 mg/L), we measured fluorescence by flow cytometry. Controls included *Proteus mirabilis* 10195 and unstained samples of each bacterial strain. (**B**) *A. baumannii* LAC-4 WT or LAC-4 ColR strains were cultured to mid-log phase in MHII, RPMI-1640, or MHII supplemented with 25 mM sodium bicarbonate. Bacteria were incubated with Dansyl-labeled polymyxin B (30 mg/L), and fluorescence was measured by flow cytometry.

To determine if the observed structural changes to LPS affected COL affinity to the bacterium, we used colistin conjugated to a BoDipy fluorophore (Fig. 5A; Table S3) or dansyl-labeled polymyxin B (Fig. 5B; Table S4) and measured colistin and polymyxin B binding affinity by flow cytometry. For these experiments, we used *P. mirabilis* 10195 to serve as a negative control due to its intrinsic resistance to colistin. Of all the strains, PM 10195 had the lowest COL affinity, indicated by its position on the far left side of the axis, in all media conditions when stained with either BoDipy-labeled colistin (Fig. 5A) or Dansyl-labeled polymyxin B (Fig. 5B). The low binding affinity of COL to PM 10195 in all media conditions is consistent with the high COL MIC observed when PM10195 is cultured in either MHII or bicarbonate conditions. In MHII conditions, there is a reduced COL affinity of BoDipy-labeled colistin for LAC-4 ColR when compared to LAC-4 WT. This reduction in COL affinity for LAC-4 ColR is consistent with the high COL MIC observed in MHII culturing conditions. When LAC-4 ColR is cultured in bicarbonate conditions, there is a notable shift to the right, indicating higher COL binding affinity of both BoDipy-labeled COL and Dansyl-labeled PMB. Most notably, culturing LAC-4 ColR in bicarbonate conditions results in a COL binding affinity profile that is equivalent to that of the colistin-susceptible strain, LAC-4 WT. All unstained samples, which served as a baseline of reference, were positioned on the far left side of the *x-*axis, indicating low background signal (Fig. 5A and B).

### Polymyxin efficacy *in vivo*

**Fig 6:**
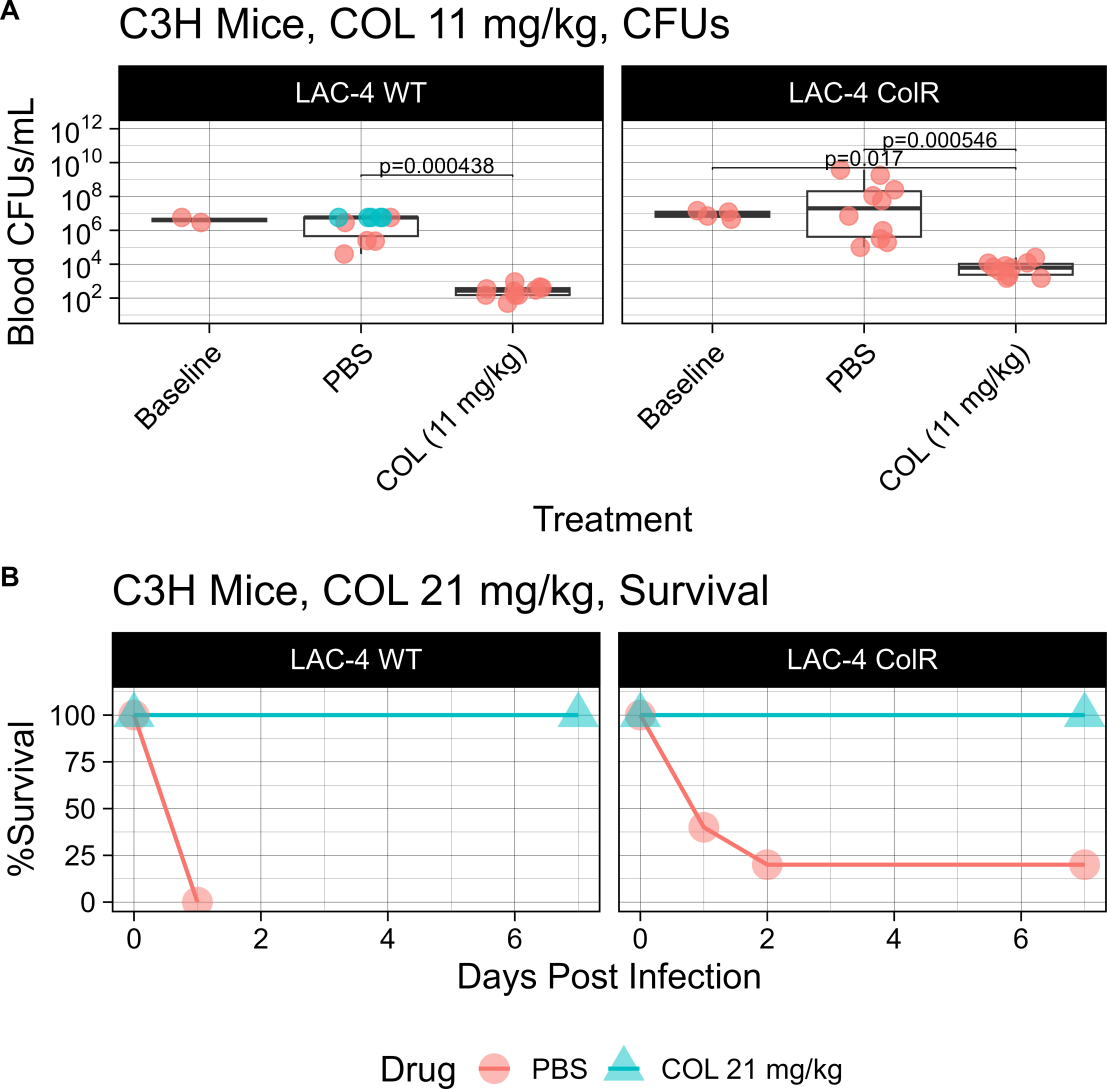
Efficacy of colistin treatment *in vivo*. (**A**) C3H mice (*n* = 12 per group) were infected with 2.1E7 CFU of LAC-4 ColR or 8.3E6 CFU of LAC-4 WT. Mice were treated subcutaneously once daily with a humanized dose of colistin (11 mg/kg/day). Blood was collected from LAC-4 WT- and LAC-4 ColR-infected mice at 14 h post infection to determine blood CFU/mL. Baseline CFUs were determined 2 h post infection. For LAC-4 WT-infected mice, 5 PBS mice succumbed to the infection before the collection time point, and so their CFUs (indicated in green) are equivalent to the counts of the high end of surviving PBS mice. Table S7 includes median CFU values. (**B**) C3H mice (*n* = 10 per group) were infected with 9.7E7 CFUs of LAC-4 ColR or 1E7 CFUs of LAC-4 WT. Mice were treated twice daily with a humanized dose of colistin (21 mg/kg/day) and monitored for survival.

To determine whether COL MICs cultured in RPMI-1640 accurately predict *in vivo* therapeutic outcomes, C3H mice were intravenously infected with *A. baumannii* strain LAC-4 WT or LAC-4 ColR (Fig. 6; Table S6). For mice infected with either strain, treatment with colistin had markedly improved survival as compared to the phosphate buffered saline (PBS) control group (WT: log rank, *P* ≤ 0.0001; ColR: log rank, *P* = 0.0126) (Fig. 6B). In LAC-4 WT-infected mice, treatment with colistin led to a significant reduction in CFU compared to the PBS control group (Mann-Whitney, *P* = 0.000438) (Fig. 6A). Notably, colistin treatment of LAC-4 ColR-infected mice resulted in a significant reduction in CFU compared to the PBS control group (Mann-Whitney, *P* = 0.000546) (Fig. 6A). Five mice infected with LAC-4 WT succumbed to infection prior to the collection time point, and so their blood could not be obtained. Because the mice succumbed to the blood infection, we assumed that the non-surviving mice had a blood CFU burden that was greater than the highest CFU burden of the surviving PBS mice. Therefore, we imputed their CFUs (green) as equivalent to the burden measured in surviving PBS mice (Fig. 6A).

**Fig 7:**
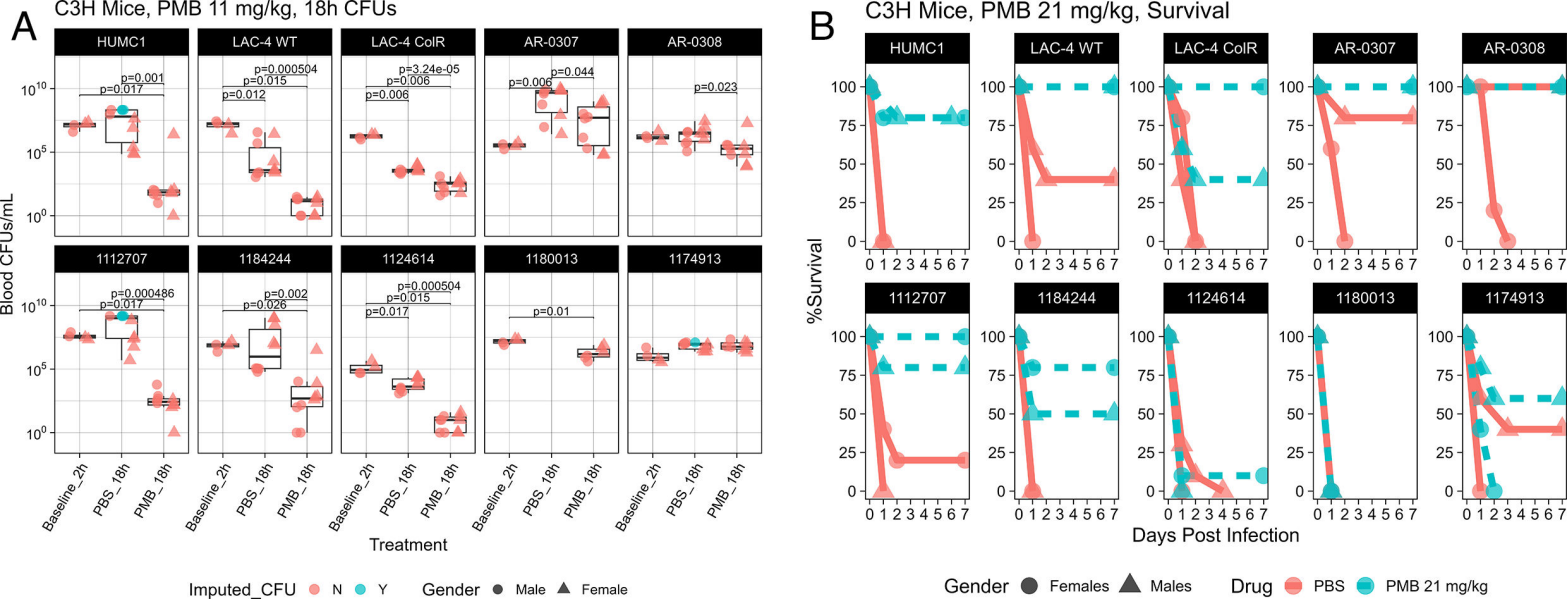
Efficacy of PMB treatment *in vivo*. (**A**) Female and male C3H mice (*n* = 5 per group) were infected with the LD100 of PMB-resistant isolates of *A. baumannii*. Mice were treated subcutaneously once with a humanized dose of PMB (11 mg/kg/day). Blood was collected from infected mice at 18 h post infection to determine blood CFU/mL. Baseline CFUs were determined 2 h post infection. For isolates 1112707 and HUMC1, four male PBS and three male PBS mice succumbed to the infection before the collection time point, and so their CFUs (indicated in green) are equivalent to the counts of the high end of surviving PBS mice. For 1174913, two male PMB mice and one male PBS mouse also succumbed before the collection time point, and so their CFUs were also imputed. (**B**) Female and male C3H mice (*n* = 5 per group) were infected with the LD100 of PMB-resistant isolates of *A. baumannii*. Mice were treated subcutaneously twice daily with a humanized dose of PMB (21 mg/kg/day) and monitored for survival.

To determine whether PMB MICs cultured in bicarbonate-containing media accurately predicted *in vivo* treatment outcomes, we selected eight PMB-resistant *A. baumannii* isolates, defined as being resistant in MHII culturing conditions, to be used in a blood infection model (Table 2). For mice infected with the PMB-susceptible strains HUMC1 and LAC-4 WT, PMB treatment led to a significant blood CFU reduction compared to the PBS group (Mann-Whitney, *P* = 0.001 and *P* = 0.000504, respectively). For mice infected with PMB-resistant strains, as defined by standard MIC testing, LAC-4 ColR, AR-0307, AR-0308, 1112707, 1184244, and 1124614, PMB treatment led to a significant reduction in CFUs compared to the PBS control group (Mann-Whitney, *P* = 0.0000324, *P* = 0.044, *P* = 0.023, *P* = 0.000486, *P* = 0.002, and *P* = 0.000504, respectively) (Fig. 7A; Table S8). There was no difference in blood CFUs between PMB and PBS groups infected with the PMB-resistant isolate 1174913. For isolate 1180013, no PBS mice survived to the collection time point, and so we were unable to collect any blood to quantify CFUs. For HUMC1, 1112707, and 1174913, not all PBS mice survived to the collection time point, and so their CFUs were imputed (green) as being equivalent to the highest CFU of surviving PBS mice. Paralleling the CFU data, PMB treatment in mice infected with LAC-4 ColR, AR-0307, AR-0308, 1112707, and 1184244 led to significantly improved survival compared to the PBS group (log rank, *P* = 0.005, *P* = 0.005, *P* = 0.011, *P* < 0.001, and *P* < 0.0001, respectively) (Fig. 7B). There was no difference in survival outcome between the PMB and PBS groups for mice infected with 1124614, 1180013, or 1174913. PMB treatment led to significantly improved survival in mice infected with the PMB-susceptible strains HUMC1 and LAC-4 WT (log rank, *P* < 0.0001 and *P* < 0.001, respectively) (Fig. 7B).

**TABLE 2:** Summary of polymyxin MICs for strains used in the *in vivo* studies

Strain	Mutation	COL MIC (mg/L) in MHII	COL MIC (mg/L) in MHII + NaHCO_3_	PMB MIC (mg/L) in MHII	PMB MIC (mg/L) in MHII + NaHCO_3_
HUMC1	Wild type	0.5	1	<0.125	<0.125
LAC-4 WT	Wild type	0.5	2	<0.125	<0.125
LAC-4 ColR	*pmrA*	>64	2	8	<0.125
AR-0307	Unknown	>64	1	8	0.25
AR-0308	Unknown	8	0.5	4	<0.125
1112707	Unknown	16	0.5	4	<0.125
1184244	Unknown	64	1	16	<0.125
1124614	Unknown	32	0.5	8	<0.125
1180013	Unknown	>64	1	8	<0.125
1174913	Unknown	32	1	8	0.5

## DISCUSSION

Colistin remains the “last line of defense” for the treatment of highly lethal infections caused by CRAB and CRE. However, as the use of colistin has increased, so has the report of colistin-resistant strains. Here, we show that conventional MHII media used in standardized antimicrobial susceptibility assays result in a disconnect of *in vitro* activity compared to *in vivo* outcomes for polymyxins for some *A. baumannii* strains. This work suggests that colistin may be effective *in vivo* despite standard susceptibility testing predicting resistance for *A. baumannii* clinical isolates.

In *A. baumannii*, colistin resistance has been shown to be conferred via chromosomal mutations in the pmrAB two-component system, which result in the addition of PetN modifications to the LPS. Colistin resistance can also be conferred via chromosomal mutations in enzymes involved in lipid A biosynthesis, which results in the complete loss of LPS. Most recently, colistin resistance has been attributed to a mobile colistin-resistance gene (*mcr-1*) which modifies the LPS with PetN groups using a plasmid-mediated phosphoethanolamine transferase. All the aforementioned mechanisms confer colistin resistance by modifying colistin’s target molecule, LPS, thereby reducing the affinity of positively charged colistin. With regard to clinical isolates, colistin resistance due to mutations in *pmrA*, *pmrB*, or *pmrC* is more commonly identified than mutations in *lpxA*, *lpxC*, or *lpxD* (20). Notably, only recently was there the first report of plasmid-mediated mcr-1 colistin resistance in an *A. baumannii* clinical isolate (21).

Our results show that culturing colistin-resistant bacteria in MHII promotes the addition of PetN modifications to LPS that disrupt its interaction with colistin. However, culturing colistin-resistant bacteria, defined as resistant in MHII conditions, in the presence of physiologically normal amounts of bicarbonate decreases the presence of PetN modifications on LPS. When we tested colistin-resistant isolates containing mutations in the enzymes involved in lipid A biosynthesis, we found that these LPS-deficient *A. baumannii* strains maintained COL and PMB resistance in physiological bicarbonate conditions. Notably, LPS-deficient *A. baumannii* strains have been shown to demonstrate attenuated virulence *in vivo* (22). Overall, our data are consistent with the current understanding of the mechanism of action for polymyxins. However, the use of bicarbonate-containing media allows for a reduced contribution of mutations that confer modifications to LPS, consistent with the observed treatment outcomes *in vivo*.

Additionally, we showed that bicarbonate itself, and not pH, is responsible for the observed change in the colistin MIC of LAC-4 ColR. In media that did not contain bicarbonate, only non-physiologically relevant alkaline conditions affected MICs, and thus changes in pH alone cannot explain the observed *in vivo* activity.

Using bicarbonate-containing media in MICs, which is more physiologically similar to the *in vivo* blood infection environment, revealed increased activity of COL against *A. baumannii*. To determine if this increased COL activity translated to effective killing in human blood, we performed time kill assays using human whole blood. Our results were only consistent with the MICs determined in bicarbonate-containing culturing conditions and not MICs using conventional MHII media, suggesting increased COL activity in the presence of bicarbonate. Due to issues with blood coagulation in the time kill assays, we were unable to achieve interpretable data necessary to make a direct comparison to the COL MICs at 24 h post infection. Despite trying several different anticoagulants (sodium citrate, anticoagulant citrate dextrose, potassium oxalate/sodium fluoride, sodium EDTA, K3 EDTA, and mechanical defibrination), we were limited to interpretable data under 4 h post infection. This limitation provides no opportunity for rebound to occur during this reduced format. Additionally, though there is a notable 2-log reduction in viability in the untreated LAC-4 ColR control at 4 h post infection, the addition of 0.001-mg/L COL at the same time point results in a greater decrease in CFUs compared to the untreated control (Mann-Whitney, *P* = 0.0273).

In mice, colistin treatment resulted in a >2-log reduction of blood bacterial CFUs for the colistin-resistant *A. baumannii* isolate LAC-4 ColR at 14 h post infection. Despite equivalent survival rates in the colistin-treated groups, the reduction in CFUs for mice infected with LAC-4 ColR was modest compared to the CFU reduction in LAC-4 WT-infected mice. To explain the difference in *in vivo* outcome between the colistin-resistant and colistin-susceptible strains, multiple factors must be considered. Firstly, it has been shown in the literature that mice infected with two different strains, albeit with equivalent MICs, can result in dissimilar *in vivo* outcomes following treatment with antibiotics (23–27). Secondly, because the colistin-resistant strain is less virulent than the colistin-susceptible strain, the *in vivo* inoculum for LAC-4 ColR (2.1e7 CFU) requires 2.5× the amount of bacteria than the LAC-4 WT strain (8.3e6 CFU) in order to achieve a lethal infection. As a result, the 2.5-fold decrease in the ratio of antibiotic:bacteria may contribute to the difference in CFU reductions observed in mice infected with colistin-resistant and colistin-susceptible strains.

We found that, for a subset of strains, using bicarbonate-containing media for PMB MIC susceptibility testing also revealed *in vivo* activity against *A. baumannii* isolates defined as polymyxin resistant in conventional MHII MIC conditions. Not all PMB-resistant strains that have low MICs in bicarbonate-containing media resulted in *in vivo* PMB activity that was protective against *A. baumannii* blood infection. The variation of PMB efficacy among our panel of *A. baumannii* clinical isolates demonstrates that, as of yet, there is no 1:1 correlation between bicarbonate-containing MIC conditions and *in vivo* PMB susceptibility. The variation in efficacy ranged from significant *in vivo* efficacy in some strains to no observable difference from the PBS control groups. For the PMB efficacy *in vivo* studies, three of six strains with a PMB-resistant breakpoint interpretation in cation adjusted Mueller-Hinton broth (CAMHB), but susceptible in bicarbonate-containing media, had >1-log median CFU reduction at 18 h post infection and a survival improvement (*A. baumannii* strains LAC-4 ColR, 1112707, and 1184244) in both male and female infected mice. For strains that were found to be susceptible in CAMHB and also bicarbonate-containing media, PMB treatment also resulted in a >1-log median CFU reduction and a survival improvement as well (*A. baumannii* strains HUMC1 and LAC-4 WT). These results support that our hypothesis is not restricted to only the LAC-4 ColR strain.

Differences in *in vivo* outcome between males and females were observed in strains AR-0307 and AR-0308. Male mice infected with AR-0307 or AR-0308 showed reduced blood CFUs following PMB treatment; however, survival experiments with male mice did not result in a lethal infection. In contrast, PMB treatment in females infected with either strain had significantly reduced blood CFUs and significantly improved survival compared to the PBS group. This discrepancy between genders is consistent with literature that demonstrated gender differences in *A. baumannii* infections in mice. Luna et al. observed that female mice were approximately twofold more sensitive to LAC-4 ColR in an *A. baumannii* blood infection (18). In our studies, the LD100 for males and females did not differ for most strains with the exception of 1112707, where the LD100 for females was 2× lower than males.

Remarkably, despite a greater than 2-log median CFU reduction in PMB-treated mice compared to the PBS control, PMB treatment in mice infected with *A. baumannii* 1124614 did not result in survival. The discrepancy between PMB-treated mice with low bacterial density but a lethal survival outcome can most likely be attributed to early induction of a systemic sepsis response that was not reversible by subsequent antibacterial therapy. In *A. baumannii* bloodstream infections in mice, lethality is driven by the interaction between bacterial LPS and host TLR4-mediated hyperinflammation (22). Although CFU enumeration seems to suggest bacterial clearance following PMB treatment, the initial high blood bacterial density achieved early in the infection may have been sufficient to trigger sepsis and lead to TLR4-mediated hyperinflammation and lethality. Additionally, it has been shown that host fate is dependent on innate effector-microbial interactions during *A. baumannii* bacteremia (28). Therefore, as the contribution of inflammation to lethality cannot be quantified by CFU enumeration experiments, further studies may elucidate the complex interactions between the isolates used in this study and the innate effectors that determine bacterial density and lethality in *A. baumannii* blood infections. These results highlight that microbiology endpoints do not always correlate with survival, and therefore, it is critical to evaluate both endpoints.

Although PMB susceptibility tests in bicarbonate-containing culturing conditions indicated PMB susceptibility, PMB treatment did not result in reduced CFUs or improved survival for mice infected with *A. baumannii* 1180013 or 1174913. Further studies are needed to explain these discrepancies. These results only further highlight that more biology needs to be understood before our observations can be applied in the clinic. Until we can explain the discrepancies between the protective efficacy that we have observed in this study, we are currently unable to predict which low MICs will be treatable and which will be untreatable. Additionally, further studies should be done to determine how the shifts in polymyxin susceptibility observed in our blood infection model translate to tissue infections.

In summary, we have shown that media containing bicarbonate, which are more physiologically representative of the host environment, accurately predicted the *in vivo* efficacy of polymyxins in *A. baumannii* blood infection for some isolates. Notably, using conventional MHII culturing conditions for antimicrobial susceptibility testing failed to identify the *in vivo* activity of polymyxins against LAC-4 ColR, 1184244, and 1112707 *A*. *baumannii* blood infections in mice. Although further work is necessary to explain strain-to-strain variability of PMB efficacy, these results show that modifying culturing conditions to better reflect the host environment can enhance the predictive power of antimicrobial susceptibility tests, which may help find solutions to the antibiotic-resistance crisis. Moreover, these results indicate that some PDR strains of *A. baumannii* may be treatable with PMB irrespective of clinical laboratory reporting of susceptibility testing in rich media, offering hope for treatment of some of these deadly infections. More fundamentally, these results underscore the importance of using physiologically representative media to enable a more accurate prediction of *in vivo* efficacy for some antibiotics and to better identify promising solutions to the antibiotic-resistance crisis.

## MATERIALS AND METHODS

### Bacterial culture

Bacteria used in the study are listed in Table S9. Working solutions of bacteria were prepared using frozen stocks of *A. baumannii* strains, as previously published (29), by inoculating a fresh overnight culture in tryptic soy broth (TSB) and incubating at 37°C/200 rpm, or by streaking on fresh tryptic soy agar (TSA) plates and incubating at 37°C. The overnight broth culture was diluted 1:100 and then subcultured in MHII, RPMI-1640, or MHII supplemented with 25 mM sodium bicarbonate at 37°C/200 rpm for 3 h and then adjusted until the culture reached an OD_600_ of 0.5. For MICs and time kill assays, bacterial cultures were adjusted to a 0.5-McFarland standard via the direct colony suspension method.

### MIC protocol

For *A. baumannii*, an MIC of ≤2 mg/L corresponds to a EUCAST susceptible and a CLSI intermediate (CLSI does not have a susceptible breakpoint for colistin) breakpoint interpretation (6, 18). Both EUCAST and CLSI agree that >2 mg/L corresponds to a resistant breakpoint. We will use the EUCAST definitions of susceptible and resistant throughout this article. Unless otherwise indicated, the standard broth microdilution method following CLSI guidelines was used to determine MICs (8). The media used for the MIC assays performed in this study were MHII (BD Biosciences, catalog no. 90000–602), RPMI-1640 (Gibco, catalog no. 11875119), and MHII supplemented with sodium bicarbonate (Sigma-Aldrich, catalog no. S5761) (at final concentration of 25 mM sodium bicarbonate, NaHCO_3_). For the RPMI-1640 components colistin MICs, each individual nutrient component was separately added to standard MHII at the final concentration reported by the manufacturer (Table S1). Colistin (Sigma, catalog no. C4461) and polymyxin B (Xellia Pharmaceuticals) were diluted in deionized water to make a fresh stock before each experiment.

Briefly, 100 µL of media was added to the wells of a 96-well plate in columns 2–10. Column 11 served as a positive growth control and contained only bacteria and media. Column 12 served as the sterility control and contained only culture media without bacteria. Next, 200 µL of a 2× antibiotic working solution was added to the wells in column 1. Twofold serial dilutions of the antibiotic were performed through column 10. Next, 100 µL of a 1 × 10^6^ CFU/mL working solution of bacteria was added to each of the wells in columns 1–11. The inoculum concentration was confirmed by plating serial dilutions on TSA plates. MIC plates were incubated at 35°C ± 2°C, and results were recorded at 24 h. MIC experiments were done in duplicate.

### Characterization of lipid A species by MALDI

Lipid A was isolated from LAC-4 WT and LAC-4 ColR, followed by MALDI-TOF mass spectrometry in the negative-ion mode as previously described (30).

### Fluorescent labeling of polymyxins and flow cytometry binding assays

Fluorescently labeled colistin was prepared as described by the manufacturer (Thermo Fisher Scientific). Briefly, colistin was labeled with BoDipy FL NHS Ester (Thermo Fisher Scientific, catalog no. D2184). One hundred microliters of BoDipy NHS ester compound (10 mg/mL in dimethyl sulfoxide), 250 µL of colistin (10 mg/mL), and 650 µL sodium bicarbonate (0.2 M, pH 8.5) were incubated at 37°C for 2 h. A Float-A-Lyser G2 dialysis device (Spectrum, catalog no. 08–607-022) with a molecular weight cutoff of 0.5 kDa was used to remove excess fluorophores not bound to colistin. Dialysis was carried out overnight in 4°C sterile distilled water, which was changed four times.

From an overnight culture, bacteria were subcultured until mid-log phase in MHII, RPMI-1640, or MHII + 25 mM NaHCO_3_. Once adjusted to an OD_600_ of 0.5, 55 µL of bacteria was added to a 96-well flat-bottom 0.22-µm filter plate and incubated with either BoDipy-labeled colistin (0.1 mg/L) for 30 minutes at 37°C or Dansyl-labeled polymyxin B (Sigma-Aldrich, catalog no. SBR00029) (30 mg/L) for 30 minutes at room temperature. For BoDipy-labeled colistin experiments, labeled BoDipy was diluted to a 1% solution using unlabeled colistin (0.01 mg/mL). The cells were then washed three times with respective media to remove any excess colistin not bound to the bacteria. The amount of colistin attached to the bacteria was determined by flow cytometry by measuring the fluorescence of 200-µL samples (10000 events). A BD Accuri C6 Plus flow cytometer was used to analyze the BoDipy-labeled colistin samples, and a BD LSR II was used to analyze the Dansyl-labeled polymyxin B samples. Controls included *Proteus mirabilis* 10195 and unstained samples of each bacterial strain. All data were analyzed using FlowJo version 10.8.

### Human blood time kill assay

The bacterial inoculum was prepared as described in bacteria culture methods. Time kill assays were performed in round-bottom 96-well plates. Bacteria was cultured in human whole blood containing K2 EDTA as the anticoagulant (Innovative Research, catalog no. IGMSCD1WBK2E25ML). Colistin was added to duplicate wells of either LAC-4 WT or LAC-4 ColR at 10, 1, 0.1, 0.01, and 0.001 mg/L. Plates were incubated at 37°C, and samples were collected at 0, 1, and 4 h post incubation. The bacterial burden at each time point was determined by plating serial dilutions on TSA plates and incubating overnight at 37°C.

### Intravenous infection

*A. baumannii* LAC-4 WT, LAC-4 ColR, and HUMC1 frozen stocks were prepared as described in previous works (18, 29). Frozen stocks of bacteria were thawed and diluted in PBS to adjust the bacterial density as needed for infection. The inoculum for all other infecting strains was freshly prepared from subcultures of bacteria cultured to mid-log phase. Separate groups of mice were used for the survival and CFU collection experiments. Male C3HeB/FeJ mice, 8–12 weeks old, were infected with the LD100 of LAC-4 WT or LAC-4 ColR via tail vein injection (Table S5). For the colistin LAC-4 ColR CFU experiment and all PMB experiments, male C3HeB/FeJ mice 7–8 weeks old and female C3HeB/FeJ mice 9–10 weeks old were infected with the LD100 via tail vein injections (Table S5). Inoculum bacterial density was confirmed by plating serial dilutions on TSA plates and incubating overnight at 37°C.

### Antibiotic treatments

Human equivalent dosing of polymyxin B was done as previously described (31). Briefly, colistin sulfate salt (Sigma-Aldrich C4461) or polymyxin B (Xellia Pharmaceuticals) was diluted in deionized water to make a stock concentration of 10 mg/mL before each experiment. Treatment was initiated at 2 h post infection, and mice were treated subcutaneously twice daily with 11 and 10 mg/kg at 0 and 12 h daily, for 2 days (or 42 h). For CFU experiments, mice were administered only a single drug treatment (11 mg/kg) to avoid excess drug carryover when plating serial dilutions.

### Blood bacterial burden

Mice assigned to the CFU collection group were collected at 2 and 14 h after infection, anesthetized intraperitoneally with 150 µL of a 30 mg/mL ketamine and 3 mg/mL xylazine solution and heparinized intraperitoneally with 20–30 µL (200 to 300 units ,United States Pharmacopeia grade). A terminal cardiac puncture was performed to collect at least 500 µL of blood. Serial dilutions of the blood samples were plated on TSA plates before incubation at 37°C overnight. Colonies were counted after 24 h, and the CFUs were calculated.

### Statistics

Gene expression and bacterial burden were compared using the Mann-Whitney test. For survival studies, time to death was compared using the log-rank test. *P* values of <0.05 were considered significant.

## Data Availability

All data are available in the main text or the supplemental material. The genome sequences for the LAC-4 WT and LAC-4 ColR strains are available at the National Center for Biotechnology Information (accession numbers NZ_CP007712 and PRJNA1023962, respectively).

## SUPPLEMENTAL MATERIAL

Supplemental material aac.00725-24-s0001.docx Supplemental methods, Fig. S1, and Tables S1 to S9.

ASM does not own the copyrights to Supplemental Material that may be linked to, or accessed through, an article. The authors have granted ASM a non-exclusive, world-wide license to publish the Supplemental Material files. Please contact the corresponding author directly for reuse.
