# Supplementary material for: Polymyxins retain in vitro activity and in vivo efficacy against “resistant” Acinetobacter baumannii strains when tested in physiological conditions
Source: Antimicrob Agents Chemother. 2024 Sep 6;68(10):e00725-24. doi: 10.1128/aac.00725-24 (PMC11459914; doi:10.1128/aac.00725-24)
Supplement: Supplemental material — Supplemental methods, Fig. S1, and Tables S1 to S9. [file aac.00725-24-s0001.docx]

# Title: Polymyxins retain *in vitro* activity and *in vivo* efficacy against “resistant” *Acinetobacter baumannii* strains when tested in physiological conditions

**Authors: Jennifer Rubio^1^, Jun Yan^1^, Sarah Miller^1^, Jiaqi Cheng^1^, Rachel Li^1^, Zac Builta^1^, Kari Aoyagi^4^, Mark Fisher^4^, Rosemary She^2^, Brad Spellberg^3^, and Brian Luna^1*^**

**Affiliations:**

^1^Department of Molecular Microbiology and Immunology, Keck School of Medicine of USC, Los Angeles, CA

^2^Department of Pathology, Keck School of Medicine of USC, Los Angeles, CA

^3^Los Angeles General Medical Center, Los Angeles, CA

^4^Department of Pathology, University of Utah, Salt Lake City, UT

*Correspondence to: Brian Luna, [brian.luna@usc.edu](mailto:brian.luna@usc.edu), USC Keck School of Medicine, 1441 Eastlake Ave, NTT 6419, Los Angeles, CA 90033.

# Supplemental Materials

**Methods**

**Whole genome sequencing**

The genome sequences of LAC-4 WT and LAC-4 ColR were determined using Illumina sequencing. Microbial DNA was extracted using Zymo Quick-DNA Bacterial miniprep kit (Genesee Scientific, catalog no. 11-321). CLC Genomics Workbench was used to perform variant analysis. Raw sequence data from the ColR strain was aligned to the LAC-4 WT strain (Accession number: NZ_CP007712) published in NCBI.

# Supplemental Fig. 1 - Whole genome sequencing of LAC-4 ColR. Genome sequences of LAC-4 WT and LAC-4 ColR were determined using Illumina sequencing, and raw sequence data from LAC-4 ColR was aligned to the LAC-4 WT strain published in NCBI. Whole genome sequencing revealed a single variation between WT and ColR: a single point mutation in *pmrA* in LAC-4 ColR.


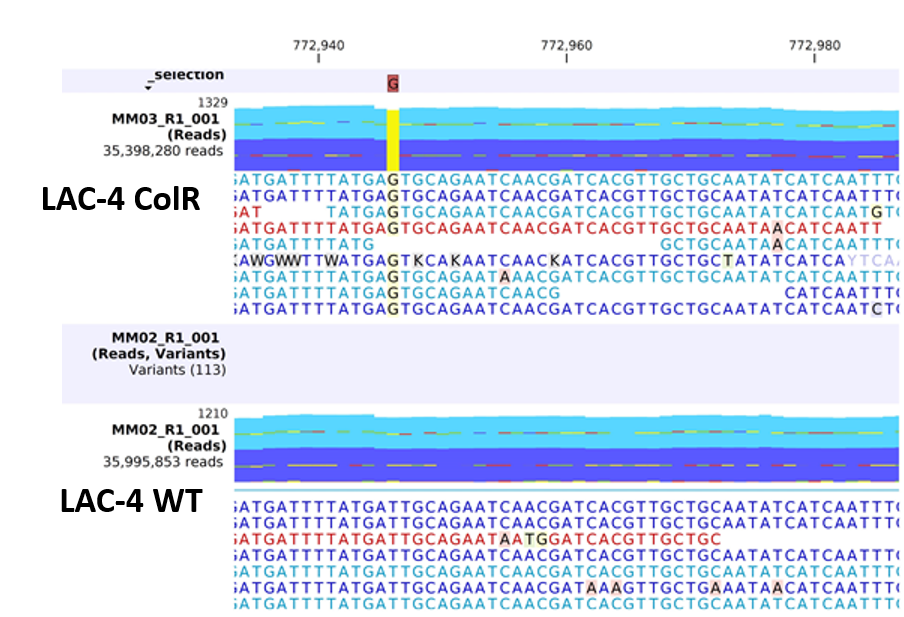


#

**Supplemental Table 1 - Colistin MICs in MHII supplemented with RPMI-1640 nutrients.** The row shaded in gray indicates the only media component that was found to affect the LAC-4 ColR COL MIC.

|  | **COL MIC (mg/L)** | |
| --- | --- | --- |
| **Media Condition** | **LAC-4 WT** | **LAC-4 ColR** |
| MHII | 0.5 | >64 |
| RPMI-1640 | 2 | 2 |
| Amino acids | | |
| MHII + 0.133mM Glycine | 0.25 | >64 |
| MHII + 1.15mM L-Arginine | 0.25 | 64 |
| MHII + 0.379mM L-Asparagine | 0.25 | >64 |
| MHII + 0.150mM L-Aspartic acid | 0.25 | >64 |
| MHII + 0.208mM L-Cysteine-2HCl | 0.25 | >64 |
| MHII + 0.136mM L-Glutamic acid | 0.25 | >64 |
| MHII + 2.05mM L-Glutamine | 0.5 | 32 |
| MHII + 0.097mM L-Histidine | 0.5 | 64 |
| MHII + 0.153mM L-Hydroxyproline | 0.5 | 32 |
| MHII + 0.382mM L-Isoleucine | 8 | 64 |
| MHII + 0.382mM L-Leucine | 0.25 | >64 |
| MHII + 0.219mM L-Lysine hydrochloride | 0.5 | 32 |
| MHII + 0.101mM L-Methionine | 0.25 | >64 |
| MHII + 0.091mM L-Phenylalanine | 0.25 | >64 |
| MHII + 0.174mM L-Proline | 0.25 | >64 |
| MHII + 0.286mM L-Serine | 0.25 | >64 |
| MHII + 0.168mM L-Threonine | 0.25 | >64 |
| MHII + 0.025mM L-Tryptophan | 0.25 | >64 |
| MHII + 0.111mM L-Tyrosine disodium salt dihydrate | 0.25 | >64 |
| MHII + 0.171mM L-Valine | 0.125 | >64 |
| Vitamins | | |
| MHII + 8.20E-04mM D-Biotin | 0.25 | >64 |
| MHII + 0.021mM Choline chloride | 0.125 | >64 |
| MHII + 5.2E-4mM Calcium-D-pantothenate | 0.125 | 64 |
| MHII + 2.27E-03mM Folic acid | 4 | 32 |
| MHII + 8.20E-03mM Niacinamide | 0.25 | >64 |
| MHII + 7.30E-03mM Para-aminobenzoic acid | 0.125 | >64 |
| MHII + 4.85E-03mM Pyridoxine hydrochloride | 0.25 | >64 |
| MHII + 5.32E-04mM Riboflavin | 0.25 | >64 |
| MHII + 2.97E-03mM Thiamine hydrochloride | 0.125 | >64 |
| MHII + 3.69E-06mM Vitamin B12 | 0.25 | >64 |
| MHII + 0.194mM i-Inositol | 0.25 | 32 |
| Inorganic salts | | |
| MHII + 0.424mM Calcium nitrate tetrahydrate | 0.5 | >64 |
| MHII + 0.41mM Magnesium sulfate | 0.125 | >64 |
| MHII + 5.33mM Potassium chloride | 0.25 | >64 |
| MHII + 25mM Sodium bicarbonate | 0.5 | 0.5 |
| MHII + 103mM Sodium chloride | 0.5 | >64 |
| MHII + 5.63mM Sodium phosphate dibasic anhydrous | 0.125 | >64 |
| Other components | | |
| MHII + 11.1mM D-Glucose | 0.25 | >64 |
| MHII + 3.26E-03mM Glutathione (reduced) | 0.125 | >64 |
| MHII + 0.013mM Phenol red | 0.5 | 64 |

# Supplemental Table 2 - Polymyxin MICs of ColR isolates in MHII or MHII supplemented with sodium bicarbonate

| **Strain** | **Mutation** | **COL MIC (mg/L) in MHII** | **COL MIC (mg/L) in MHII + NaHCO3** | **PMB MIC (mg/L) in MHII** | **PMB MIC (mg/L) in MHII + NaHCO3** |
| --- | --- | --- | --- | --- | --- |
| LAC-4 ColR | *pmrA* | >64 | 2 | 8 | <0.125 |
| C8 | *pmrB* | 64 | 1 | 64 | 1 |
| C14 | *pmrB, pmrC* | 32 | 2 | 8 | 1 |
| D773 mcr-1 | *mcr-1* | >64 | 1 | 4 | 0.5 |
| SM1536 mcr-1 | *mcr-1* | >64 | 1 | 8 | 2 |
| 17978 mcr-1 | *mcr-1* | 64 | 8 | 8 | 8 |
| 19606R | *lpxA* | >64 | >64 | >64 | >64 |
| AL1833 | *lpxC* | >64 | 16 | 64 | 64 |
| AL1834 | *lpxC* | >64 | 32 | 64 | 64 |
| AL1842 | *lpxC* | >64 | 16 | >64 | >64 |
| AL1843 | *lpxC* | >64 | >64 | >64 | >64 |
| AL1844 | *lpxA* | >64 | >64 | >64 | >64 |
| AL1845 | *lpxA* | >64 | >64 | >64 | >64 |
| AL1846 | *lpxA* | >64 | >64 | >64 | >64 |
| AL1847 | *lpxA* | >64 | >64 | >64 | >64 |
| AL1848 | *lpxA* | >64 | >64 | >64 | >64 |
| AL1849 | *lpxA* | >64 | >64 | >64 | >64 |
| AL1851 | *lpxA* | >64 | >64 | >64 | >64 |
| AL1852 | *lpxD* | >64 | >64 | >64 | >64 |
| ARUP_A1 | Unknown | 64 | 0.25 | 4 | 0.125 |
| ARUP_A6 | Unknown | >64 | 0.25 | 32 | 0.125 |
| ARUP_A8 | Unknown | 64 | 8 | 8 | 8 |
| ARUP_A10 | Unknown | >64 | 0.5 | 16 | 0.125 |
| ARUP_A11 | Unknown | >64 | 0.25 | 16 | 0.25 |
| ARUP_A13 | Unknown | >64 | 0.25 | 32 | 0.125 |
| ARUP_A22 | Unknown | >64 | 0.25 | 32 | 0.125 |
| ARUP_A23 | Unknown | >64 | 2 | 64 | 0.5 |
| ARUP_A28 | Unknown | 16 | 0.25 | 64 | 0.125 |
| ARUP_A30 | Unknown | >64 | 0.25 | 4 | 0.125 |
| AR-0307 | Unknown | >64 | 1 | 8 | 0.25 |
| AR-0308 | Unknown | 8 | 0.5 | 4 | <0.125 |
| 1112707 | Unknown | 16 | 0.5 | 4 | <0.125 |
| 1184244 | Unknown | 64 | 1 | 16 | <0.125 |
| 1124614 | Unknown | 32 | 0.5 | 8 | <0.125 |
| 1180013 | Unknown | >64 | 1 | 8 | <0.125 |
| 1174913 | Unknown | 32 | 1 | 8 | 0.5 |
| 1064048 | Unknown | 32 | 2 | - | - |
| 1071864 | Unknown | 64 | 4 | - | - |
| 1083037 | Unknown | 64 | 1 | - | - |
| 1083246 | Unknown | >64 | 1 | - | - |
| 1083383 | Unknown | >64 | 1 | - | - |
| 1099731 | Unknown | >64 | 1 | - | - |
| 1105437 | Unknown | >64 | 0.5 | - | - |
| 1125675 | Unknown | >64 | 1 | - | - |
| 1127911 | Unknown | >64 | 1 | - | - |
| 1172798 | Unknown | 8 | 2 | - | - |
| 1174945 | Unknown | >64 | 2 | - | - |
| 1178685 | Unknown | 16 | 1 | - | - |
| 1180949 | Unknown | 64 | 4 | - | - |
| 1181046 | Unknown | >64 | 1 | - | - |
| 1185713 | Unknown | 64 | 1 | - | - |
| 1188882 | Unknown | 64 | 1 | - | - |
| ARUP_A3 | Unknown | 16 | 0.25 | - | - |
| ARUP_A4 | Unknown | 4 | 0.25 | - | - |
| ARUP_A5 | Unknown | 16 | 0.125 | - | - |
| ARUP_A9 | Unknown | 8 | 0.5 | - | - |
| ARUP_A14 | Unknown | 16 | 1 | - | - |
| ARUP_A16 | Unknown | 8 | 0.5 | - | - |
| ARUP_A18 | Unknown | 8 | 1 | - | - |
| ARUP_A19 | Unknown | 16 | 0.25 | - | - |
| ARUP_A20 | Unknown | 32 | 0.5 | - | - |
| ARUP_A26 | Unknown | 64 | 0.25 | - | - |
| ARUP_A29 | Unknown | >64 | 0.13 | - | - |

**“ - ” indicates not tested**

# Supplemental Table 3 - Affinity of BoDipy-labeled colistin to ColR bacteria

| **Strain** | **Condition** | **MFI (mean fluorescence intensity) of BoDipy** | |
| --- | --- | --- | --- |
|  |  | **MHII** | **RPMI** |
| AB LAC-4 ColR | Unstained | 247 | 21 |
| AB LAC-4 ColR | Stained | 938 | 1225 |
| AB LAC-4 WT | Unstained | 121 | 23.8 |
| AB LAC-4 WT | Stained | 10556 | 1230 |
| PM 10195 | Unstained | 17.1 | 9.29 |
| PM 10195 | Stained | 30.1 | 149 |

# Supplemental Table 4 - Affinity of Dansyl-labeled polymyxin B to ColR bacteria

| **Strain** | **Condition** | **MFI (mean fluorescence intensity)** | | |
| --- | --- | --- | --- | --- |
|  |  | **MHII** | **RPMI** | **MHII + NaHCO3** |
| AB LAC-4 WT | Unstained | 16.1 | 15.3 | 16 |
| PM 10195 | Unstained | 12.6 | 11.2 | 13.3 |
| AB LAC-4 ColR | Stained | 1189 | 3782 | 2067 |
| AB LAC-4 WT | Stained | 2403 | 2296 | 2283 |
| PM 10195 | Stained | 175 | 483 | 201 |

# Supplemental Table 5 - Summary of LD100 virulence screening

| **Strain** | **Female** | | | **Male** | | |
| --- | --- | --- | --- | --- | --- | --- |
|  | **LD100 (CFU/mouse)** | **Sub-LD100 (CFU/mouse)** | **CFU/mL** | **LD100 (CFU/mouse)** | **Sub-LD100 (CFU/mouse)** | **CFU/mL** |
| AR-0307 | 3.07E+08 | 2.65E+08 | 6.14E+08 | 4.23E+08 | 3.58E+08 | 8.46E+08 |
| AR-0308 | 4.83E+08 | - | 9.66E+08 | 4.83E+08 | 1.30E+08 | 9.66E+08 |
| 1112707 | 6.83E+07 | - | 2.73E+08 | 1.25E+08 | 3.83E+07 | 5.00E+08 |
| 1184244 | 6.73E+07 | - | 2.69E+08 | 5.70E+07 | 1.77E+07 | 2.28E+08 |
| 1124614 | 5.92E+07 | - | 2.37E+08 | 5.92E+07 | 2.08E+07 | 2.37E+08 |
| 1180013 | 3.20E+08 | - | 6.40E+08 | 3.20E+08 | 1.40E+08 | 6.40E+08 |
| 1174913 | 5.00E+08 | - | 1.00E+09 | 5.00E+08 | 2.69E+08 | 1.00E+09 |

# Supplemental Table 6 - Efficacy of colistin treatment *in vivo*

| **Strain** | **Mouse** | **Treatment** | **Gender** | **Time point (hours)** | **CFU/mL** |
| --- | --- | --- | --- | --- | --- |
| LAC-4 WT | 1 | Baseline | Male | 2 | 2.90E+06 |
|  | 2 | Baseline | Male | 2 | 5.75E+06 |
|  | 1 | PBS | Male | 14 | 2.30E+05 |
|  | 2 | PBS | Male | 14 | 2.45E+05 |
|  | 3 | PBS | Male | 14 | 4.00E+04 |
|  | 4 | PBS | Male | 14 | 2.90E+06 |
|  | 5 | PBS | Male | 14 | 5.75E+06 |
|  | 6 | PBS | Male | 14 | 5.75E+06* |
|  | 7 | PBS | Male | 14 | 5.75E+06* |
|  | 8 | PBS | Male | 14 | 5.75E+06* |
|  | 9 | PBS | Male | 14 | 5.75E+06* |
|  | 10 | PBS | Male | 14 | 5.75E+06* |
|  | 1 | COL (11 mg/kg/day) | Male | 14 | 5.00E+01 |
|  | 2 | COL (11 mg/kg/day) | Male | 14 | 9.00E+02 |
|  | 3 | COL (11 mg/kg/day) | Male | 14 | 1.50E+02 |
|  | 4 | COL (11 mg/kg/day) | Male | 14 | 3.50E+02 |
|  | 5 | COL (11 mg/kg/day) | Male | 14 | 1.50E+02 |
|  | 6 | COL (11 mg/kg/day) | Male | 14 | 4.00E+02 |
|  | 7 | COL (11 mg/kg/day) | Male | 14 | 4.50E+02 |
|  | 8 | COL (11 mg/kg/day) | Male | 14 | 1.50E+02 |
|  | 9 | COL (11 mg/kg/day) | Male | 14 | 3.00E+02 |
|  | 10 | COL (11 mg/kg/day) | Male | 14 | 2.50E+02 |
| LAC-4 ColR | 1 | Baseline | Male | 2 | 1.45E+07 |
|  | 2 | Baseline | Male | 2 | 1.20E+07 |
|  | 3 | Baseline | Female | 2 | 7.00E+06 |
|  | 4 | Baseline | Female | 2 | 4.50E+06 |
|  | 1 | PBS | Male | 14 | 1.10E+08 |
|  | 2 | PBS | Male | 14 | 2.50E+08 |
|  | 3 | PBS | Male | 14 | 1.00E+05 |
|  | 4 | PBS | Male | 14 | 9.50E+05 |
|  | 5 | PBS | Male | 14 | 1.95E+05 |
|  | 6 | PBS | Female | 14 | 5.70E+07 |
|  | 7 | PBS | Female | 14 | 3.80E+09 |
|  | 8 | PBS | Female | 14 | 1.80E+09 |
|  | 9 | PBS | Female | 14 | 3.15E+05 |
|  | 10 | PBS | Female | 14 | 6.95E+06 |
|  | 1 | COL 11 mg/kg | Male | 14 | 1.20E+04 |
|  | 2 | COL 11 mg/kg | Male | 14 | 8.00E+03 |
|  | 3 | COL 11 mg/kg | Male | 14 | 1.15E+04 |
|  | 4 | COL 11 mg/kg | Male | 14 | 2.50E+04 |
|  | 5 | COL 11 mg/kg | Male | 14 | 6.50E+03 |
|  | 6 | COL 11 mg/kg | Female | 14 | 1.50E+03 |
|  | 7 | COL 11 mg/kg | Female | 14 | 2.00E+03 |
|  | 8 | COL 11 mg/kg | Female | 14 | 4.00E+03 |
|  | 9 | COL 11 mg/kg | Female | 14 | 6.00E+03 |
|  | 10 | COL 11 mg/kg | Female | 14 | 1.50E+03 |

**“ * ” indicates value was imputed**

# Supplemental Table 7 - Median CFU values for colistin efficacy studies

| **Strain** | **Treatment** | **Gender** | **Time point (hours)** | **Median CFU/mL** |
| --- | --- | --- | --- | --- |
| LAC-4 WT | Baseline | Male | 2 | 4.33E+06 |
|  | PBS | Male | 14 | 5.75E+06 |
|  | COL 11 mg/kg | Male | 14 | 2.75E+02 |
| LAC-4 ColR | Baseline | Male and Female | 2 | 9.50E+06 |
|  | PBS | Male and Female | 14 | 3.20E+07 |
|  | COL 11 mg/kg | Male and Female | 14 | 6.25E+03 |

# Supplemental Table 8 - Efficacy of polymyxin treatment *in vivo*

| **Strain** | **Mouse** | **Treatment** | **Gender** | **Time point (hours)** | **CFU/mL** |
| --- | --- | --- | --- | --- | --- |
| HUMC1 | 1 | Baseline | Female | 2 | 2.30E+07 |
|  | 2 | Baseline | Female | 2 | 1.70E+07 |
|  | 3 | Baseline | Male | 2 | 4.00E+06 |
|  | 4 | Baseline | Male | 2 | 1.35E+07 |
|  | 1 | PBS | Female | 18 | 2.35E+05 |
|  | 2 | PBS | Female | 18 | 4.35E+07 |
|  | 3 | PBS | Female | 18 | 8.50E+04 |
|  | 4 | PBS | Female | 18 | 7.00E+04 |
|  | 5 | PBS | Female | 18 | 8.00E+06 |
|  | 6 | PBS | Male | 18 | 9.30E+07 |
|  | 7 | PBS | Male | 18 | 2.1E+08 |
|  | 8 | PBS | Male | 18 | 2.1E+08* |
|  | 9 | PBS | Male | 18 | 2.1E+08* |
|  | 10 | PBS | Male | 18 | 2.1E+08* |
|  | 1 | PMB 21 mg/kg | Female | 18 | 6.00E+01 |
|  | 2 | PMB 21 mg/kg | Female | 18 | 1.10E+02 |
|  | 3 | PMB 21 mg/kg | Female | 18 | 2.50E+06 |
|  | 4 | PMB 21 mg/kg | Female | 18 | 8.00E+01 |
|  | 5 | PMB 21 mg/kg | Female | 18 | 1.00E+00 |
|  | 6 | PMB 21 mg/kg | Male | 18 | 1.00E+01 |
|  | 7 | PMB 21 mg/kg | Male | 18 | 4.00E+01 |
|  | 8 | PMB 21 mg/kg | Male | 18 | 5.00E+01 |
|  | 9 | PMB 21 mg/kg | Male | 18 | 9.00E+01 |
|  | 10 | PMB 21 mg/kg | Male | 18 | 1.10E+02 |
| LAC-4 WT | 1 | Baseline | Female | 2 | 2.90E+06 |
|  | 2 | Baseline | Female | 2 | 1.50E+07 |
|  | 3 | Baseline | Male | 2 | 2.70E+07 |
|  | 4 | Baseline | Male | 2 | 1.85E+07 |
|  | 1 | PBS | Female | 18 | 3.55E+03 |
|  | 2 | PBS | Female | 18 | 2.50E+03 |
|  | 3 | PBS | Female | 18 | 2.70E+06 |
|  | 4 | PBS | Female | 18 | 4.00E+03 |
|  | 5 | PBS | Female | 18 | 1.85E+04 |
|  | 6 | PBS | Male | 18 | 1.10E+03 |
|  | 7 | PBS | Male | 18 | 2.20E+03 |
|  | 8 | PBS | Male | 18 | 5.05E+05 |
|  | 9 | PBS | Male | 18 | 3.70E+06 |
|  | 10 | PBS | Male | 18 | 2.60E+03 |
|  | 1 | PMB 21 mg/kg | Female | 18 | 1.00E+00 |
|  | 2 | PMB 21 mg/kg | Female | 18 | 3.00E+01 |
|  | 3 | PMB 21 mg/kg | Female | 18 | 1.00E+01 |
|  | 4 | PMB 21 mg/kg | Female | 18 | 1.00E+00 |
|  | 5 | PMB 21 mg/kg | Female | 18 | 2.00E+01 |
|  | 6 | PMB 21 mg/kg | Male | 18 | 1.00E+00 |
|  | 7 | PMB 21 mg/kg | Male | 18 | 1.00E+00 |
|  | 8 | PMB 21 mg/kg | Male | 18 | 2.00E+01 |
|  | 9 | PMB 21 mg/kg | Male | 18 | 2.00E+01 |
|  | 10 | PMB 21 mg/kg | Male | 18 | 3.00E+01 |
| LAC-4 ColR | 1 | Baseline | Female | 2 | 2.30E+06 |
|  | 2 | Baseline | Female | 2 | 2.45E+06 |
|  | 3 | Baseline | Male | 2 | 1.45E+06 |
|  | 4 | Baseline | Male | 2 | 1.00E+06 |
|  | 1 | PBS | Female | 18 | 1.05E+04 |
|  | 2 | PBS | Female | 18 | 3.03E+03 |
|  | 3 | PBS | Female | 18 | 1.00E+04 |
|  | 4 | PBS | Female | 18 | 4.48E+03 |
|  | 5 | PBS | Female | 18 | 3.63E+03 |
|  | 6 | PBS | Male | 18 | 2.58E+03 |
|  | 7 | PBS | Male | 18 | 4.25E+03 |
|  | 8 | PBS | Male | 18 | 2.51E+03 |
|  | 9 | PBS | Male | 18 | 1.97E+03 |
|  | 10 | PBS | Male | 18 | 3.15E+03 |
|  | 1 | PMB 21 mg/kg | Female | 18 | 4.60E+02 |
|  | 2 | PMB 21 mg/kg | Female | 18 | 6.00E+01 |
|  | 3 | PMB 21 mg/kg | Female | 18 | 7.70E+02 |
|  | 4 | PMB 21 mg/kg | Female | 18 | 3.40E+02 |
|  | 5 | PMB 21 mg/kg | Female | 18 | 3.70E+02 |
|  | 6 | PMB 21 mg/kg | Male | 18 | 7.00E+01 |
|  | 7 | PMB 21 mg/kg | Male | 18 | 1.60E+02 |
|  | 8 | PMB 21 mg/kg | Male | 18 | 4.00E+01 |
|  | 9 | PMB 21 mg/kg | Male | 18 | 3.30E+02 |
|  | 10 | PMB 21 mg/kg | Male | 18 | 1.28E+03 |
| AR-0307 | 1 | Baseline | Female | 2 | 5.55E+05 |
|  | 2 | Baseline | Female | 2 | 3.00E+05 |
|  | 3 | Baseline | Male | 2 | 4.15E+05 |
|  | 4 | Baseline | Male | 2 | 1.70E+05 |
|  | 1 | PBS | Female | 18 | 8.20E+07 |
|  | 2 | PBS | Female | 18 | 7.00E+09 |
|  | 3 | PBS | Female | 18 | 6.70E+09 |
|  | 4 | PBS | Female | 18 | 2.60E+06 |
|  | 5 | PBS | Female | 18 | 1.00E+10 |
|  | 6 | PBS | Male | 18 | 5.70E+08 |
|  | 7 | PBS | Male | 18 | 4.00E+09 |
|  | 8 | PBS | Male | 18 | 5.70E+09 |
|  | 9 | PBS | Male | 18 | 9.50E+06 |
|  | 10 | PBS | Male | 18 | 9.20E+09 |
|  | 1 | PMB 21 mg/kg | Female | 18 | 8.50E+08 |
|  | 2 | PMB 21 mg/kg | Female | 18 | 7.00E+04 |
|  | 3 | PMB 21 mg/kg | Female | 18 | 1.00E+09 |
|  | 4 | PMB 21 mg/kg | Female | 18 | 5.50E+08 |
|  | 5 | PMB 21 mg/kg | Female | 18 | 6.00E+04 |
|  | 6 | PMB 21 mg/kg | Male | 18 | 5.80E+07 |
|  | 7 | PMB 21 mg/kg | Male | 18 | 2.00E+05 |
|  | 8 | PMB 21 mg/kg | Male | 18 | 1.05E+08 |
|  | 9 | PMB 21 mg/kg | Male | 18 | 4.50E+07 |
|  | 10 | PMB 21 mg/kg | Male | 18 | 1.40E+06 |
| AR-0308 | 1 | Baseline | Female | 2 | 7.65E+05 |
|  | 2 | Baseline | Female | 2 | 4.35E+06 |
|  | 3 | Baseline | Male | 2 | 1.80E+06 |
|  | 4 | Baseline | Male | 2 | 1.28E+06 |
|  | 1 | PBS | Female | 18 | 3.00E+06 |
|  | 2 | PBS | Female | 18 | 2.75E+06 |
|  | 3 | PBS | Female | 18 | 9.60E+06 |
|  | 4 | PBS | Female | 18 | 9.65E+05 |
|  | 5 | PBS | Female | 18 | 2.80E+07 |
|  | 6 | PBS | Male | 18 | 3.95E+06 |
|  | 7 | PBS | Male | 18 | 4.95E+05 |
|  | 8 | PBS | Male | 18 | 1.16E+05 |
|  | 9 | PBS | Male | 18 | 3.55E+06 |
|  | 10 | PBS | Male | 18 | 6.40E+05 |
|  | 1 | PMB 21 mg/kg | Female | 18 | 7.30E+04 |
|  | 2 | PMB 21 mg/kg | Female | 18 | 1.90E+07 |
|  | 3 | PMB 21 mg/kg | Female | 18 | 9.00E+03 |
|  | 4 | PMB 21 mg/kg | Female | 18 | 2.95E+05 |
|  | 5 | PMB 21 mg/kg | Female | 18 | 7.50E+03 |
|  | 6 | PMB 21 mg/kg | Male | 18 | 6.25E+04 |
|  | 7 | PMB 21 mg/kg | Male | 18 | 1.90E+05 |
|  | 8 | PMB 21 mg/kg | Male | 18 | 3.75E+05 |
|  | 9 | PMB 21 mg/kg | Male | 18 | 3.55E+05 |
| 1112707 | 1 | Baseline | Female | 2 | 2.19E+07 |
|  | 2 | Baseline | Female | 2 | 3.03E+07 |
|  | 3 | Baseline | Male | 2 | 7.90E+07 |
|  | 4 | Baseline | Male | 2 | 3.80E+07 |
|  | 1 | PBS | Female | 18 | 7.00E+08 |
|  | 2 | PBS | Female | 18 | 5.80E+06 |
|  | 3 | PBS | Female | 18 | 3.10E+07 |
|  | 4 | PBS | Female | 18 | 2.45E+07 |
|  | 5 | PBS | Female | 18 | 4.95E+05 |
|  | 6 | PBS | Male | 18 | 1.52E+09 |
|  | 7 | PBS | Male | 18 | 1.52E+09* |
|  | 8 | PBS | Male | 18 | 1.52E+09* |
|  | 9 | PBS | Male | 18 | 1.52E+09* |
|  | 10 | PBS | Male | 18 | 1.52E+09* |
|  | 1 | PMB 21 mg/kg | Female | 18 | 4.00E+02 |
|  | 2 | PMB 21 mg/kg | Female | 18 | 1.00E+00 |
|  | 3 | PMB 21 mg/kg | Female | 18 | 1.00E+02 |
|  | 4 | PMB 21 mg/kg | Female | 18 | 1.50E+02 |
|  | 5 | PMB 21 mg/kg | Female | 18 | 1.50E+02 |
|  | 6 | PMB 21 mg/kg | Male | 18 | 6.00E+03 |
|  | 7 | PMB 21 mg/kg | Male | 18 | 7.70E+02 |
|  | 8 | PMB 21 mg/kg | Male | 18 | 2.10E+02 |
|  | 9 | PMB 21 mg/kg | Male | 18 | 4.80E+02 |
|  | 10 | PMB 21 mg/kg | Male | 18 | 3.30E+02 |
| 1184244 | 1 | Baseline | Female | 2 | 1.52E+07 |
|  | 2 | Baseline | Female | 2 | 7.65E+06 |
|  | 3 | Baseline | Male | 2 | 8.30E+06 |
|  | 4 | Baseline | Male | 2 | 2.30E+06 |
|  | 1 | PBS | Female | 18 | 1.07E+09 |
|  | 2 | PBS | Female | 18 | 8.05E+06 |
|  | 3 | PBS | Female | 18 | 9.65E+08 |
|  | 4 | PBS | Female | 18 | 3.05E+08 |
|  | 5 | PBS | Female | 18 | 1.11E+07 |
|  | 6 | PBS | Male | 18 | 1.14E+05 |
|  | 7 | PBS | Male | 18 | 1.12E+05 |
|  | 8 | PBS | Male | 18 | 1.11E+05 |
|  | 9 | PBS | Male | 18 | 6.15E+04 |
|  | 10 | PBS | Male | 18 | 1.06E+05 |
|  | 1 | PMB 21 mg/kg | Female | 18 | 6.00E+02 |
|  | 2 | PMB 21 mg/kg | Female | 18 | 7.00E+02 |
|  | 3 | PMB 21 mg/kg | Female | 18 | 7.50E+03 |
|  | 4 | PMB 21 mg/kg | Female | 18 | 4.00E+02 |
|  | 5 | PMB 21 mg/kg | Female | 18 | 3.20E+06 |
|  | 6 | PMB 21 mg/kg | Male | 18 | 1.50E+02 |
|  | 7 | PMB 21 mg/kg | Male | 18 | 1.00E+00 |
|  | 8 | PMB 21 mg/kg | Male | 18 | 1.00E+02 |
|  | 9 | PMB 21 mg/kg | Male | 18 | 1.05E+04 |
|  | 10 | PMB 21 mg/kg | Male | 18 | 1.00E+00 |
| 1124614 | 1 | Baseline | Female | 2 | 1.50E+05 |
|  | 2 | Baseline | Female | 2 | 4.50E+05 |
|  | 3 | Baseline | Male | 2 | 5.00E+04 |
|  | 4 | Baseline | Male | 2 | 5.00E+04 |
|  | 1 | PBS | Female | 18 | 5.00E+03 |
|  | 2 | PBS | Female | 18 | 2.15E+04 |
|  | 3 | PBS | Female | 18 | 2.30E+04 |
|  | 4 | PBS | Female | 18 | 9.00E+03 |
|  | 5 | PBS | Female | 18 | 2.05E+04 |
|  | 6 | PBS | Male | 18 | 1.23E+03 |
|  | 7 | PBS | Male | 18 | 2.50E+03 |
|  | 8 | PBS | Male | 18 | 2.91E+03 |
|  | 9 | PBS | Male | 18 | 3.50E+03 |
|  | 10 | PBS | Male | 18 | 1.68E+03 |
|  | 1 | PMB 21 mg/kg | Female | 18 | 1.00E+00 |
|  | 2 | PMB 21 mg/kg | Female | 18 | 4.00E+01 |
|  | 3 | PMB 21 mg/kg | Female | 18 | 2.00E+01 |
|  | 4 | PMB 21 mg/kg | Female | 18 | 1.00E+00 |
|  | 5 | PMB 21 mg/kg | Female | 18 | 1.00E+01 |
|  | 6 | PMB 21 mg/kg | Male | 18 | 1.00E+00 |
|  | 7 | PMB 21 mg/kg | Male | 18 | 2.00E+01 |
|  | 8 | PMB 21 mg/kg | Male | 18 | 1.00E+01 |
|  | 9 | PMB 21 mg/kg | Male | 18 | 1.00E+01 |
|  | 10 | PMB 21 mg/kg | Male | 18 | 1.00E+00 |
| 1180013 | 1 | Baseline | Female | 2 | 2.05E+07 |
|  | 2 | Baseline | Female | 2 | 2.30E+07 |
|  | 3 | Baseline | Male | 2 | 1.25E+07 |
|  | 4 | Baseline | Male | 2 | 8.00E+06 |
|  | 1 | PMB 21 mg/kg | Female | 18 | 4.50E+06 |
|  | 2 | PMB 21 mg/kg | Female | 18 | 7.80E+06 |
|  | 3 | PMB 21 mg/kg | Female | 18 | 2.00E+06 |
|  | 4 | PMB 21 mg/kg | Male | 18 | 8.20E+05 |
|  | 5 | PMB 21 mg/kg | Male | 18 | 1.17E+06 |
|  | 6 | PMB 21 mg/kg | Male | 18 | 4.00E+05 |
| 1174913 | 1 | Baseline | Female | 2 | 3.50E+05 |
|  | 2 | Baseline | Female | 2 | 5.50E+05 |
|  | 3 | Baseline | Male | 2 | 4.95E+06 |
|  | 4 | Baseline | Male | 2 | 1.10E+06 |
|  | 1 | PBS | Female | 18 | 2.35E+06 |
|  | 2 | PBS | Female | 18 | 3.50E+06 |
|  | 3 | PBS | Female | 18 | 4.65E+06 |
|  | 4 | PBS | Female | 18 | 8.60E+06 |
|  | 5 | PBS | Female | 18 | 2.50E+06 |
|  | 6 | PBS | Male | 18 | 1.25E+07 |
|  | 7 | PBS | Male | 18 | 1.00E+07 |
|  | 8 | PBS | Male | 18 | 8.50E+06 |
|  | 9 | PBS | Male | 18 | 1.10E+07 |
|  | 10 | PBS | Male | 18 | 1.25E+07* |
|  | 1 | PMB 21 mg/kg | Female | 18 | 1.35E+07 |
|  | 2 | PMB 21 mg/kg | Female | 18 | 1.10E+07 |
|  | 3 | PMB 21 mg/kg | Female | 18 | 2.00E+06 |
|  | 4 | PMB 21 mg/kg | Female | 18 | 2.95E+06 |
|  | 5 | PMB 21 mg/kg | Female | 18 | 4.05E+06 |
|  | 6 | PMB 21 mg/kg | Male | 18 | 5.50E+06 |
|  | 7 | PMB 21 mg/kg | Male | 18 | 2.20E+07 |
|  | 8 | PMB 21 mg/kg | Male | 18 | 6.00E+06 |

**“ * ” indicates value was imputed**

# Supplemental Table 9 - Summary of bacterial strains used in this study

| **Strains** | **Description** | **Reference** |
| --- | --- | --- |
| *A. baumannii* HUMC1 | Clinical isolate | [33] |
| *A. baumannii* LAC-4 WT | Clinical isolate | [18] |
| *A. baumannii* LAC-4 ColR | Colistin-resistant mutant of LAC-4 WT; *pmrA* | [18] |
| *P. mirabilis* 10195 | Clinical isolate | This study |
| *A. baumannii* C8 | Colistin-resistant; *pmrB* | [10] |
| *A. baumannii* C14 | Colistin-resistant mutant; *pmrB, pmrC* | [10] |
| *A. baumannii* SM1536 (mcr-1) | Colistin-resistant mutant; *mcr-1* | [34] |
| *A. baumannii* ATCC 17978 (mcr-1) | Colistin-resistant mutant; *mcr-1* | [34] |
| *A. baumannii* D773 (mcr-1) | Colistin-resistant mutant; *mcr-1* | [34] |
| *A. baumannii* 19606R | Colistin-resistant mutant; *lpxA* | [35] |
| *A. baumannii* AL1833 | Colistin-resistant mutant; *lpxC* | [35] |
| *A. baumannii* AL1834 | Colistin-resistant mutant; *lpxC* | [35] |
| *A. baumannii* 1842 | Colistin-resistant mutant; *lpxC* | [35] |
| *A. baumannii* 1843 | Colistin-resistant mutant; *lpxC* | [35] |
| *A. baumannii* 1844 | Colistin-resistant mutant; *lpxA* | [35] |
| *A. baumannii* 1845 | Colistin-resistant mutant; *lpxA* | [35] |
| *A. baumannii* 1846 | Colistin-resistant mutant; *lpxA* | [35] |
| *A. baumannii* 1847 | Colistin-resistant mutant; *lpxA* | [35] |
| *A. baumannii* 1848 | Colistin-resistant mutant; *lpxA* | [35] |
| *A. baumannii* 1849 | Colistin-resistant mutant; *lpxA* | [35] |
| *A. baumannii* 1851 | Colistin-resistant mutant; *lpxA* | [35] |
| *A. baumannii* 1852 | Colistin-resistant mutant; *lpxD* | [35] |
| *A. baumannii* ARUP_A1 | Colistin-resistant clinical isolate | This study |
| *A. baumannii* ARUP_A6 | Colistin-resistant clinical isolate | This study |
| *A. baumannii* ARUP_A8 | Colistin-resistant clinical isolate | This study |
| *A. baumannii* ARUP_A10 | Colistin-resistant clinical isolate | This study |
| *A. baumannii* ARUP_A11 | Colistin-resistant clinical isolate | This study |
| *A. baumannii* ARUP_A13 | Colistin-resistant clinical isolate | This study |
| *A. baumannii* ARUP_A22 | Colistin-resistant clinical isolate | This study |
| *A. baumannii* ARUP_A23 | Colistin-resistant clinical isolate | This study |
| *A. baumannii* ARUP_A28 | Colistin-resistant clinical isolate | This study |
| *A. baumannii* ARUP_A30 | Colistin-resistant clinical isolate | This study |
| *A. baumannii* AR-0307 | Colistin-resistant clinical isolate | This study |
| *A. baumannii* AR-0308 | Colistin-resistant clinical isolate | This study |
| *A. baumannii* 1112707 | Colistin-resistant clinical isolate | This study |
| *A. baumannii* 1184244 | Colistin-resistant clinical isolate | This study |
| *A. baumannii* 1124614 | Colistin-resistant clinical isolate | This study |
| *A. baumannii* 1180013 | Colistin-resistant clinical isolate | This study |
| *A. baumannii* 1174913 | Colistin-resistant clinical isolate | This study |
| *A. baumannii* 1064048 | Colistin-resistant clinical isolate | This study |
| *A. baumannii* 1071864 | Colistin-resistant clinical isolate | This study |
| *A. baumannii* 1083037 | Colistin-resistant clinical isolate | This study |
| *A. baumannii* 1083246 | Colistin-resistant clinical isolate | This study |
| *A. baumannii* 1083383 | Colistin-resistant clinical isolate | This study |
| *A. baumannii* 1099731 | Colistin-resistant clinical isolate | This study |
| *A. baumannii* 1105437 | Colistin-resistant clinical isolate | This study |
| *A. baumannii* 1125675 | Colistin-resistant clinical isolate | This study |
| *A. baumannii* 1127911 | Colistin-resistant clinical isolate | This study |
| *A. baumannii* 1172798 | Colistin-resistant clinical isolate | This study |
| *A. baumannii* 1174945 | Colistin-resistant clinical isolate | This study |
| *A. baumannii* 1178685 | Colistin-resistant clinical isolate | This study |
| *A. baumannii* 1180949 | Colistin-resistant clinical isolate | This study |
| *A. baumannii* 1185713 | Colistin-resistant clinical isolate | This study |
| *A. baumannii* 1188882 | Colistin-resistant clinical isolate | This study |
| *A. baumannii* ARUP_A3 | Colistin-resistant clinical isolate | This study |
| *A. baumannii* ARUP_A4 | Colistin-resistant clinical isolate | This study |
| *A. baumannii* ARUP_A5 | Colistin-resistant clinical isolate | This study |
| *A. baumannii* ARUP_A9 | Colistin-resistant clinical isolate | This study |
| *A. baumannii* ARUP_A14 | Colistin-resistant clinical isolate | This study |
| *A. baumannii* ARUP_A16 | Colistin-resistant clinical isolate | This study |
| *A. baumannii* ARUP_A18 | Colistin-resistant clinical isolate | This study |
| *A. baumannii* ARUP_A19 | Colistin-resistant clinical isolate | This study |
| *A. baumannii* ARUP_A20 | Colistin-resistant clinical isolate | This study |
| *A. baumannii* ARUP_A26 | Colistin-resistant clinical isolate | This study |
| *A. baumannii* ARUP_A29 | Colistin-resistant clinical isolate | This study |

**References**

33. Luo G, Lin L, Ibrahim AS, Baquir B, Pantapalangkoor P, Bonomo RA, Doi Y, Adams MD, Russo TA, Spellberg B. 2012. Active and passive immunization protects against lethal, extreme drug resistant-Acinetobacter baumannii infection. PLoS ONE 7:e29446. https://doi.org/10.1371/journal.pone.0029446

34. Liu Y-Y, Chandler CE, Leung LM, McElheny CL, Mettus RT, Shanks RMQ, Liu J-H, Goodlett DR, Ernst RK, Doi Y. 2017. Structural modification of lipopolysaccharide conferred by mcr-1 in Gram-negative ESKAPE pathogens. Antimicrob Agents Chemother 61:e00580-17. https://doi.org/10.1128/AAC.00580-17

35. Moffatt JH, Harper M, Harrison P, Hale JDF, Vinogradov E, Seemann T, Henry R, Crane B, St Michael F, Cox AD, Adler B, Nation RL, Li J, Boyce JD. 2010. Colistin resistance in Acinetobacter baumannii is mediated by complete loss of lipopolysaccharide production. Antimicrob Agents Chemother 54:4971–4977. https://doi.org/10.1128/AAC.00834-10
